# Loss of MAGEL2 in Prader-Willi syndrome leads to decreased secretory granule and neuropeptide production

**DOI:** 10.1172/jci.insight.138576

**Published:** 2020-09-03

**Authors:** Helen Chen, A. Kaitlyn Victor, Jonathon Klein, Klementina Fon Tacer, Derek J.C. Tai, Celine de Esch, Alexander Nuttle, Jamshid Temirov, Lisa C. Burnett, Michael Rosenbaum, Yiying Zhang, Li Ding, James J. Moresco, Jolene K. Diedrich, John R. Yates, Heather S. Tillman, Rudolph L. Leibel, Michael E. Talkowski, Daniel D. Billadeau, Lawrence T. Reiter, Patrick Ryan Potts

**Affiliations:** 1 Department of Cell and Molecular Biology, St. Jude Children’s Research Hospital, Memphis, Tennessee, USA.; 2 Department of Neurology, Department of Pediatrics, and Department of Anatomy and Neurobiology, University of Tennessee Health Science Center, Memphis, Tennessee, USA.; 3 Center for Genomic Medicine, Department of Neurology, Department of Pathology, and Department of Psychiatry, Massachusetts General Hospital, Boston, Massachusetts, USA.; 4 Department of Neurology, Harvard Medical School, Boston, Massachusetts, USA.; 5 Program in Medical and Population Genetics and Stanley Center for Psychiatric Research, Broad Institute, Cambridge, Massachusetts, USA.; 6 Levo Therapeutics, Inc., Skokie, Illinois, USA.; 7 Division of Molecular Genetics, Department of Pediatrics, and Naomi Berrie Diabetes Center, Vagelos College of Physicians and Surgeons, Columbia University Irving Medical Center, New York, New York, USA.; 8 Division of Oncology Research and Schulze Center for Novel Therapeutics, Mayo Clinic, Rochester, Minnesota, USA.; 9 Department of Molecular Medicine, The Scripps Research Institute, La Jolla, California, USA.; 10 Veterinary Pathology Core, St. Jude Children’s Research Hospital, Memphis, Tennessee, USA.

**Keywords:** Cell Biology, Neuroscience, Neurodevelopment, Protein traffic, iPS cells

## Abstract

Prader-Willi syndrome (PWS) is a developmental disorder caused by loss of maternally imprinted genes on 15q11-q13, including melanoma antigen gene family member L2 (*MAGEL2*). The clinical phenotypes of PWS suggest impaired hypothalamic neuroendocrine function; however, the exact cellular defects are unknown. Here, we report deficits in secretory granule (SG) abundance and bioactive neuropeptide production upon loss of MAGEL2 in humans and mice. Unbiased proteomic analysis of *Magel2*^p*Δ*/m+^** mice revealed a reduction in components of SG in the hypothalamus that was confirmed in 2 PWS patient–derived neuronal cell models. Mechanistically, we show that proper endosomal trafficking by the MAGEL2-regulated WASH complex is required to prevent aberrant lysosomal degradation of SG proteins and reduction of mature SG abundance. Importantly, loss of MAGEL2 in mice, NGN2-induced neurons, and human patients led to reduced neuropeptide production. Thus, MAGEL2 plays an important role in hypothalamic neuroendocrine function, and cellular defects in this pathway may contribute to PWS disease etiology. Moreover, these findings suggest unanticipated approaches for therapeutic intervention.

## Abstract 2

Prader-Willi Syndrome arises in part through loss of MAGEL2-regulated secretory granule biogenesis and neuropeptide production in the hypothalamus.

## Graphical abstract

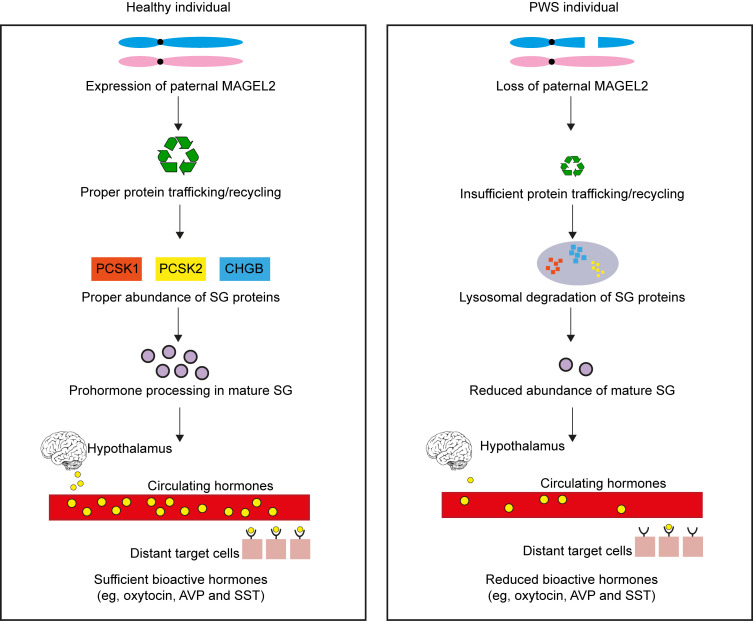

## Introduction

Prader-Willi syndrome (PWS; OMIM #176270) is a complex neurogenetic disorder that affects 1 in 15,000 children, with 400,000 cases diagnosed globally (1). PWS is a contiguous gene disorder caused by paternal loss of the maternally imprinted 15q11-q13 chromosomal region containing 6 small nucleolar RNA genes and 6 protein-coding genes (*MKRN3*, *NDN*, *NPAP1*, *SNURF-SNRPN*, and melanoma antigen gene family member L2; *MAGEL2*) (1). Genetic lesions leading to PWS can be classified into 3 major categories: deletion of paternal 15q11-q13 (65%–75% of cases), maternal uniparental disomy (UPD) (20%–30%), or imprinting defects (1%–3%) (2). Major clinical features of PWS include neonatal hypotonia and failure to thrive, intellectual and physical disabilities, endocrine dysfunctions, hyperphagia, obesity, maladaptive behaviors, increased risk of type 2 diabetes, and a higher incidence of autism spectrum disorder (ASD) (1, 2). These broad-spectrum endocrine, neurological, and behavior phenotypes have implicated a number of organ systems, including the neuroendocrine functions of the hypothalamus. Notably, genes within the PWS locus are highly expressed in the hypothalamus, including *MAGEL2* that shows strong enrichment in several hypothalamic nuclei (3–5). There is no cure or effective therapy for PWS because of the limited understanding of the underlying molecular and cellular etiology of the disease. Growth hormone replacement therapy is the only FDA-approved treatment for PWS.

Of the PWS locus genes, *MAGEL2* is particularly interesting given that paternal, de novo mutations of *MAGEL2* cause the PWS-related disorder Schaaf-Yang syndrome (SYS; OMIM #615547) (6, 7). SYS presents partially overlapping symptoms with PWS, including developmental delay, neonatal hypotonia, and hypogonadism but with a higher prevalence of ASD (8). In addition, mice with targeted deletion of *Magel2* recapitulate fundamental aspects of PWS and SYS (9, 10), including growth retardation at early stages of life, increased adult adiposity, and abnormal circadian rhythms (4, 9, 11, 12). Together, these data from both human and mouse studies make the *MAGEL2* gene an excellent candidate responsible for at least some of the clinical characteristics of PWS.

MAGEL2 is a member of the MAGE family that function as components of E3 ubiquitin ligases (13–15). The physiological role of MAGEL2 is best described using cancer cell models, where MAGEL2 partners with E3 ubiquitin ligases to facilitate protein trafficking through the retromer pathway (14–16). The retromer endosomal protein recycling pathway is an essential process that facilitates the trafficking of membrane proteins from endosomes to either the plasma membrane or the trans-Golgi network (TGN), thus preventing their trafficking to and degradation in lysosomes (17, 18). Endosomal cargos include intracellular sorting receptors such as mammalian mannose 6-phosphate receptors (19), transmembrane peptidases such as furin (20), and cell adhesion proteins such as integrin and cadherin family members (21). In support of a role for Magel2 in endocytic recycling, a study using *Magel2*-deficient mice and tumor cell line models revealed that cell surface recycling of the leptin receptor is impaired (22).

Endosomal cargos are first recognized by the conserved retromer complex (VPS35, VPS26, and VPS29) and associated proteins that facilitate their sorting into endosomal subdomains and tubules (23, 24). Endosomal F-actin plays an important role in this process, including establishment of endosomal subdomains, membrane remodeling, and vesicle scission to compartmentalize cargos (25). The WASH regulatory complex (SHRC), composed of WASH, FAM21, CCDC53, SWIP, and Strumpellin, promotes F-actin nucleation by recruiting the Arp2/3 complex onto endosomes (26). Previously, we showed that WASH activity is regulated by MAGEL2 and its associated proteins within the MAGE-L2–USP7–TRIM27 complex that promotes WASH activation and downstream actin nucleation through nondegradative, K63-linked polyubiquitination of WASH (15, 16). How alteration of this pathway in the context of PWS and in relevant tissue types (the hypothalamus) and cell types (neurons) has been unclear. Additionally, whether defects in these pathways are observable in PWS patient–derived cells has not been determined.

In this study, we used unbiased, quantitative proteomics to identify proteins in the mouse hypothalamus whose abundances are altered by loss of *Magel2*. This analysis revealed that the secretory granule (SG) abundance and neuropeptide production pathway are dramatically impaired following the loss of *Magel2*. Previous studies observed hormonal imbalance in PWS patients and *Magel2* paternal truncation (*Magel2^pΔ/m+^*) mice (1, 4, 11, 27–30). However, the cellular defects that lead to these changes have not been elucidated. Here, we show that the abundances of several SG proteins are decreased because of impaired endosomal protein trafficking leading to lysosomal degradation. These decreases resulted in reduced SG number and downstream neuropeptide production and release. A major challenge of studying neurogenetic disorders like PWS is the availability of in vitro models that accurately reflect the disease state. Here, we developed human induced pluripotent stem cell (iPSC) and dental pulp stem cell (DPSC) neuronal culture systems to model PWS. Importantly, we show that SG abundance and neuropeptide production are impaired in both isogenic PWS deletion iPSC-neurons and patient-derived PWS DPSC-neurons. Thus, in both human and mouse PWS models, we identify a previously unappreciated role for MAGEL2 in regulating SG abundance and neuropeptide production that may play an important role in disease pathogenesis.

## Results

### Endogenous expression of Magel2 protein is restricted to the hypothalamus and amygdala in the mouse brain.

**Figure 1:**
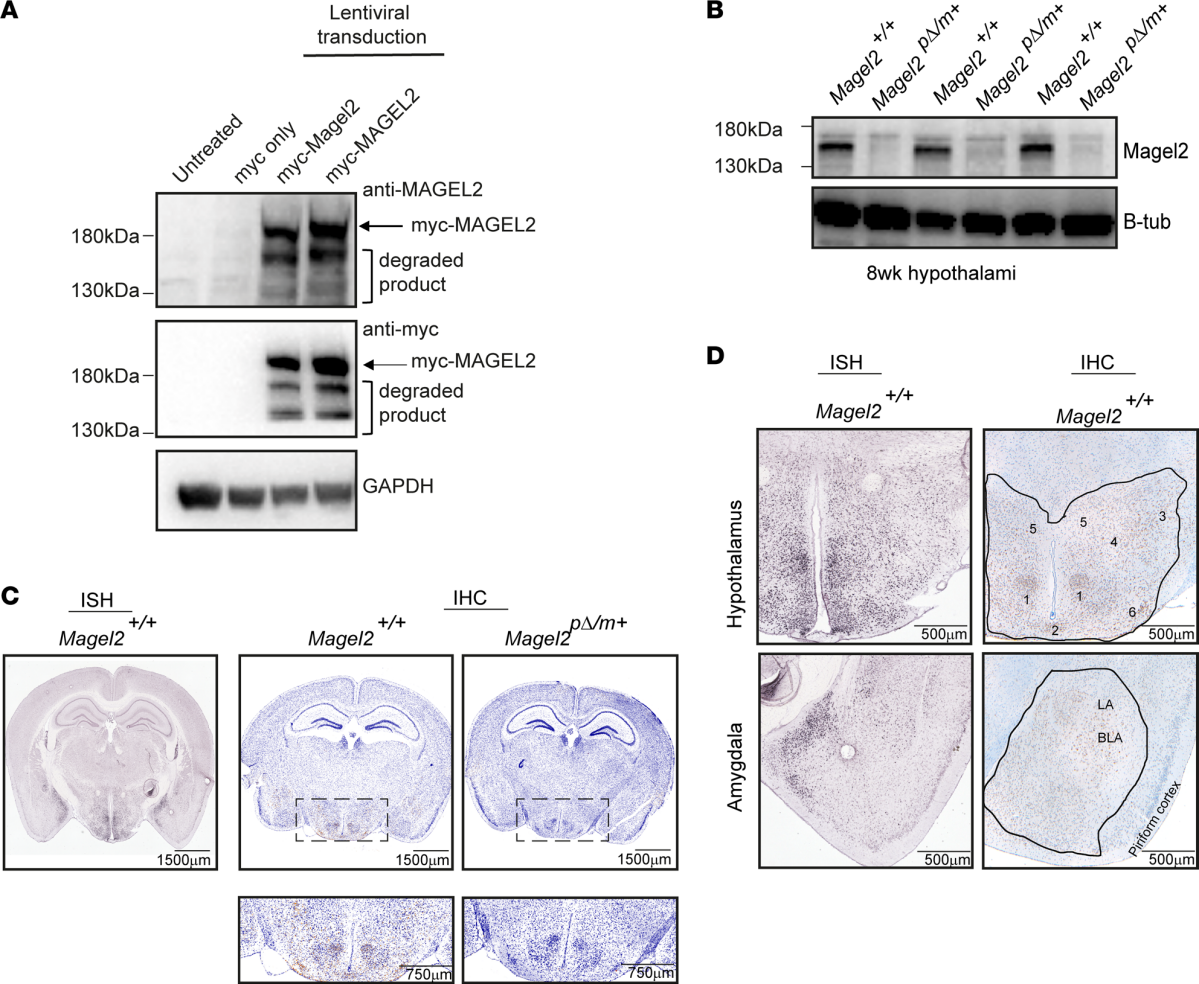
MAGEL2 is expressed in specific brain regions. (**A**) Western blot analysis detected expression of myc-tagged human and mouse MAGEL2 in HEK293 cells (ATCC, CRL-1573) by lentiviral transduction. Arrow marks myc-tagged exogenous protein; degraded protein products were also detected. GAPDH served as loading control. (**B**) Western blot analysis confirmed loss of Magel2 in 8-week-old *Magel2*^p*Δ*/m+^** mouse hypothalamuses compared with *Magel2^+/+^*. β-Tubulin served as loading control. (**C**) IHC of an 8-week-old *Magel2^+/+^* mouse brain compared with in situ hybridization (ISH) of *Magel2^+/+^* mouse brain by the Allen Brain Atlas. Note absence of brown staining in null animals. (**D**) IHC of an 8-week-old *Magel2^+/+^* mouse at the hypothalamus and amygdala as outlined in black. Brown staining indicates Magel2. Anatomical landmarks within the hypothalamus are listed as 1) ventromedial hypothalamic nucleus (VMH), 2) arcuate hypothalamic nucleus (ARH), 3) lateral hypothalamic nucleus (LHA), 4) anterior hypothalamic nucleus (AHN), 5) paraventricular hypothalamic nucleus (PVN), and 6) tuberal nucleus (TU). LA, lateral region; BLA, basolateral region.

Previous studies of MAGEL2 have relied on detection of transcript levels as a surrogate for protein expression and the use of overexpression-tagged protein for functional studies (14–16, 22, 31) because of the lack of commercial antibodies that robustly detect endogenous MAGEL2 with sufficient sensitivity and specificity. To overcome this limitation, we generated a polyclonal Magel2 antibody against amino acids 970–1284 of the mouse protein corresponding to sequences within the highly conserved MAGE homology domain. By Western blot analysis, we detected overexpressed myc-tagged mouse and human MAGEL2 in HEK293 cells (Figure 1A). Next, we used hypothalamic tissue from WT (*Magel2^+/+^*) or mice with a LacZ insertion into the C-terminal of *Magel2* that retained the N-terminal region of the gene (*Magel2^pΔ/m+^*) to perform Western blot analysis. The Magel2 polyclonal antibody robustly detected endogenous Magel2 (apparent molecular weight of ~150 kDa) in WT but not *Magel2^pΔ/m+^* animals (Figure 1B). By quantitative real-time PCR (qRT-PCR) MAGEL2 expression is highest in the adult human and mouse brain (16, 32). Thus, we performed immunohistochemistry (IHC) on 8-week-old *Magel2^pΔ/m+^* mice and control *Magel2^+/+^* littermates to determine which brain regions express Magel2 protein. As expected (3, 4, 11, 16, 32), IHC revealed immunoreactivity localized to the cytoplasm within subpopulations of neurons in the hypothalamus of *Magel2^+/+^* but not *Magel2^pΔ/m+^* mice (Figure 1, C and D; and Supplemental Figure 1A; supplemental material available online with this article; https://doi.org/10.1172/jci.insight.138576DS1). Immunoreactivity of Magel2 appeared to be confined to neurons, and no significant immunoreactivity in macroglial or microglial was observed. There were a range of staining intensities within the different hypothalamic nuclei (Figure 1D); moderate to strong immunoreactivity was detected at the VMH, ARH, and TU, while weak to moderate immunoreactivity was detected at the LHA, AHN, and PVN (Figure 1D). Interestingly, Magel2 was also detected in the amygdala (Figure 1, C and D; and Supplemental Figure 1A), with notable immunoreactivity at lateral and basolateral regions and weak staining throughout the rest of the amygdala, confirming previous findings (4). This finding is interesting given that the amygdala is an important part of the neural network that comprises the “social brain,” in which people with ASD show reduced amygdala activity (33), while SYS patients with truncating mutations in MAGEL2 have a high prevalence of ASD (7, 8). Our IHC findings were corroborated by Allen Brain Atlas ISH results showing notable *Magel2* transcript levels in the hypothalamus and amygdala (Figure 1, C and D).

### Quantitative proteomics reveals loss of SG proteins in Magel2^pΔ-/m+^ mice.

**Figure 2:**
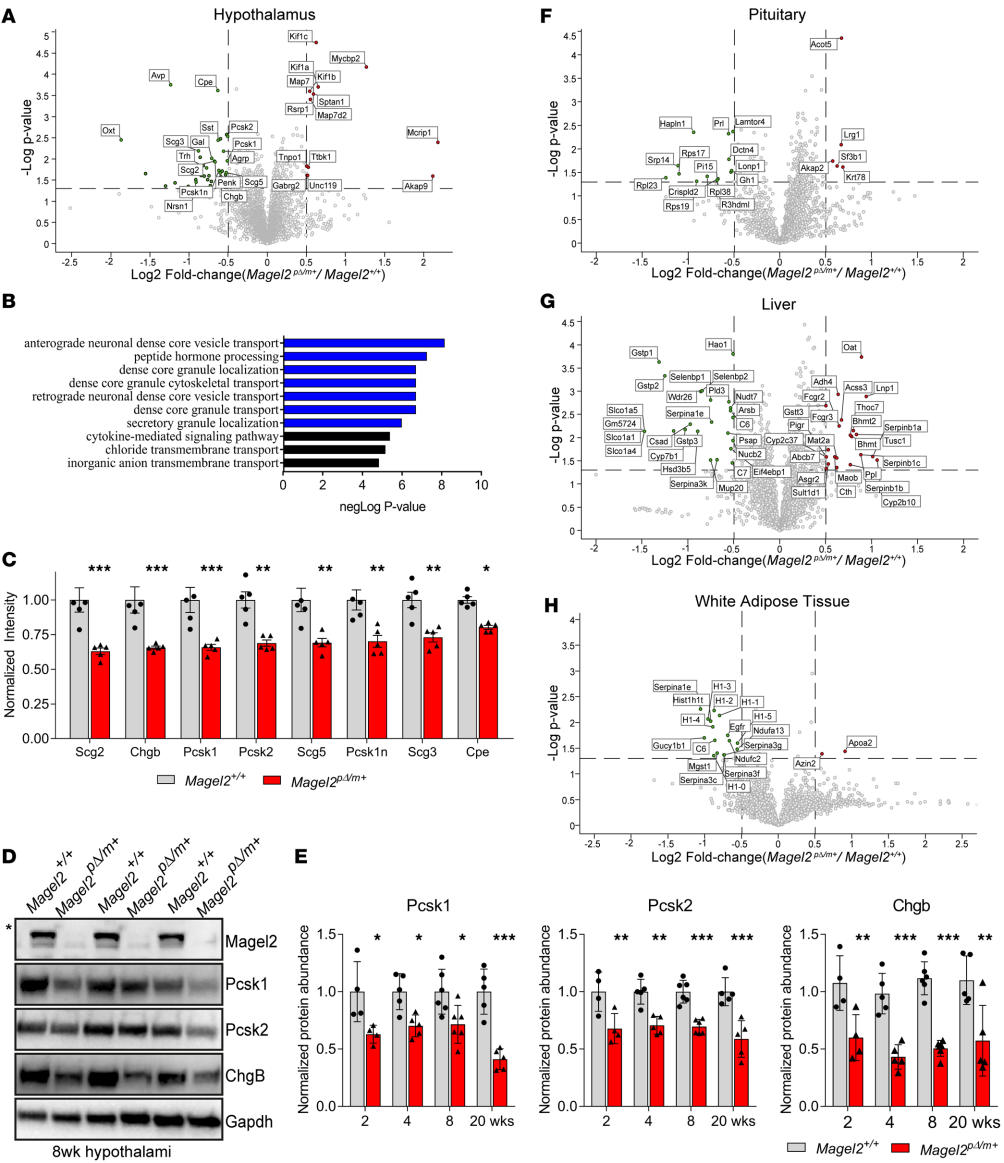
Quantitative proteomics reveals loss of SG proteins in hypothalamus of *Magel2^pΔ/m+^* mice. (**A**) Volcano plots showing proteins that are significantly changed in 8-week-old *Magel2*^p*Δ*/m+^** mouse hypothalamuses as detected by TMT. The horizontal lines denote *P* value thresholds (*P* ≤ 0.05; analyzed by unpaired, 2-tailed *t* test), and vertical lines denote log_2_ fold change thresholds (>0.5 and <–0.5), *n* = 5 per genotype. (**B**) Gene ontology categories that are enriched based on proteins with significantly decreased abundance in *Magel2*^p*Δ*/m+^** mouse hypothalamuses. (**C**) Peptide abundances of various SG proteins are significantly reduced in 8-week-old *Magel2*^p*Δ*/m+^** mouse hypothalamuses. The *Magel2*^p*Δ*/m+^** animal is normalized to averaged *Magel2^+/+^* mice; *n* = 5 per genotype. Each data point represents 1 animal, plotted as mean ± SD and indicated *P* values as analyzed by unpaired, 2-tailed *t* test with Bonferroni’s correction. (**D**) Western blot analysis confirmed reduced expression of Pcsk1, Pcsk2, and Chgb in 8-week-old *Magel2^p-/m+^* mouse hypothalamuses. Asterisks mark a nonspecific band. Gapdh served as loading control. (**E**) Quantification of Western blot analysis showed reduced expression of Pcsk1, Pcsk2, and Chgb in *Magel2*^p*Δ*/m+^** mouse hypothalamuses at 2, 4, 8, and 20 weeks; *n* > 4 per genotype. Each target protein is first normalized to Gapdh, and then the *Magel2^p-/m+^* animal is normalized to averaged *Magel2^+/+^* mice. Each data point represents 1 animal, plotted as mean ± SD and analyzed by 2-tailed *t* test with Bonferroni’s correction. (**F**–**H**) Volcano plots showing proteins that are significantly changed in *Magel2^p–/m+^* mouse (**F**) pituitary (8 weeks old), (**G**) liver (5.4 months old), and (**H**) white adipose tissue (5.4 months old), as detected by TMT. The horizontal lines denote *P* value thresholds (*P* ≤ 0.05; analyzed by unpaired, 2-tailed *t* test), and vertical lines denote log_2_ fold change thresholds (>0.5 and <–0.5). *n* = 5 per genotype. *P < 0.05 and **P < 0.01.

To gain insights into MAGEL2-regulated processes that may contribute to PWS pathology, we performed comprehensive, unbiased, quantitative tandem mass tagging (TMT) proteomics on *Magel2^pΔ/m+^* paternal loss of function mice. We analyzed hypothalamus, pituitary, adrenal, brainstem, liver, and white adipose tissues from 2- to 5-month-old *Magel2^pΔ/m+^* mice and control *Magel2^+/+^* littermates, with 2803–4986 distinct proteins being quantitated in specific tissues (Supplemental Table 1). Within the hypothalamus, only 52 proteins were significantly different, 40 downregulated and 12 upregulated, in *Magel2^pΔ/m+^* animals (Figure 2A). Our analyses are based on whole tissue, thus including both intra- and extracellular proteins. Gene ontology analysis revealed that many of the downregulated proteins (17 of 40) were components of the dense SGs involved in the processing and release of neuropeptides (Figure 2B and Supplemental Table 2). This included granins (chromogranin B [Chgb], secretogranin II [Scg2], Scg3, and Scg5), proprotein convertases and regulators (proprotein convertase subtilisin/kexin type 1 [Pcsk1], Pcsk2, carboxypeptidase E [Cpe], and Pcsk1n), and neuropeptides (vasopressin [Avp], galanin, oxytocin [Oxt], proenkephalin, somatostatin [Sst], and thyrotropin-releasing hormone) (Figure 2C). Importantly, these findings were supported using orthogonal immunoblotting assays that showed depletion of SG components in the hypothalamuses of *Magel2^pΔ/m+^* animals at 2, 4, 8, and 20 weeks of age (Figure 2, D and E; Supplemental Figure 1, B–D).

SGs are hallmarks of neuroendocrine cells and function as exocytic organelles for processing and secretion of hormones and neuropeptides (34, 35). In the regulated secretory pathway (reviewed in refs. 34, 36), hormones and neuropeptides are synthesized as larger inactive precursors in the endoplasmic reticulum. They are then packaged and processed to active peptides in SGs and secreted in a regulated manner. Granins coalesce propeptides and accessory proteins within the TGN before immature SGs emerge and undergo a maturation process that involves homotypic fusion of immature SGs, removal of missorted proteins for lysosomal degradation, vesicle acidification, and material condensation. Acidification of SGs is needed to activate processing enzymes, such as PCSK1, PCSK2, and CPE, to process prohormones into their bioactive, cleaved forms (37, 38). Mature SGs can store large quantities of active neuropeptides for days until their release upon stimulation.

Consistent with SG defects in *Magel2^pΔ/m+^* hypothalamic tissue having a functional significance, proteomics analysis of the hypothalamus revealed decreased levels of several major neuropeptides, including Oxt, Avp, and Sst (Figure 2A), that have been implicated in PWS neurobiology. Furthermore, quantitative proteomics of the pituitary (Figure 2F) showed decreased levels of growth hormone, luteinizing hormone, and prolactin, which rely on neurosecretory input from the hypothalamus via the median eminence. Given that the hypothalamus/pituitary axis has inputs to many tissues in the body and that PWS is a systemic disease, we next examined other tissues that may be indirectly affected by loss of Magel2 in the hypothalamus. Proteomic analyses of liver and white adipose tissues revealed dysregulation of a number of metabolic genes in *Magel2^pΔ/m+^* animals (Figure 2, G and H), while brainstem and adrenal tissues showed minor differences (Supplemental Figure 2, A and B). Together, these findings suggest that SG and neuropeptide abundance are altered in *Magel2^pΔ/m+^* mice.

### SG proteins are reduced in PWS disease model human iPSC-derived NGN2-induced neurons.

**Figure 3:**
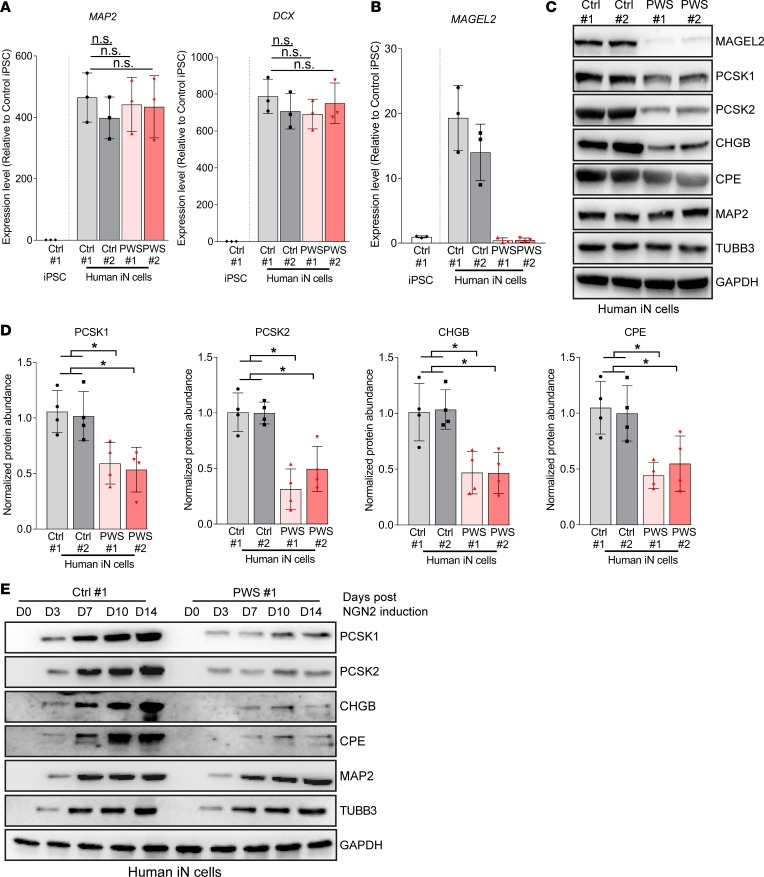
SG proteins are reduced in human iN with PWS deletion. (**A**) Transcript levels of *MAP2* and *DCX* are significantly increased in control and PWS iN at 14 days postinduction. PWS iN are normalized to averaged control iN. Each data point represents 1 induction experiment (*n* = 3), plotted with mean ± SD and analyzed by 1-way ANOVA. (**B**) Transcript levels of *MAGEL2* are significantly increased in control iN at 14 days postinduction. Each data point represents 1 induction experiment (*n* = 3), plotted as mean ± SD and analyzed by 1-way ANOVA. (**C**) Western blot analysis confirms reduced expression of PCSK1, PCSK2, CHGB, and CPE in PWS iN at 14 days postinduction. GAPDH served as loading control. (**D**) Quantification of Western blot analysis showed reduced expression of PCSK1, PCSK2, CHGB, and CPE in PWS iN. Each target protein is first normalized to GAPDH, and then PWS iN is normalized to averaged control iN. Each data point represents 1 induction experiment (*n* = 4), plotted as mean ± SD and analyzed by 1-way ANOVA; *P < 0.05. (**E**) Western blot analysis confirms reduced expression of PCSK1, PCSK2, CHGB, and CPE in PWS iN compared with control iN at 3, 7, 10, and 14 days postinduction. GAPDH served as loading control.

To elucidate whether SG protein abundances are disturbed in a human PWS neuronal disease model system, we generated human iPSCs with paternal type I PWS deletion via CRISPR/Cas9 genome editing of female MGH2069 cells. This deletion encompasses loss of genetic materials between breakpoints 1 and 3 (~6 Mb; Supplemental Figure 3A), which accounts for approximately 40% of diagnosed PWS cases (39). We generated 2 PWS deletion iPSC clones, PWS 1 and PWS 2, along with 2 control iPSC clones, Ctrl 1 and Ctrl 2, all from the same isogenic background. iPSCs were differentiated into cortex-like neurons via doxycycline-inducible expression of neurogenin 2 (NGN2) for 14 days to produce induced neurons (iNeurons, hereafter iN) (40). Robust NGN2-GFP expression could be detected 1 day after doxycycline induction, and neuronal morphology could be observed 7 days postinduction (Supplemental Figure 3B). At 14 days after NGN2 induction, qRT-PCR analysis revealed that iN expressed increased levels of endogenous neuronal markers microtubule-associated protein 2 (*MAP2*) and doublecortin (*DCX*), whose levels were elevated nearly 400- and 800-fold, respectively (Figure 3A). We noted equal levels of *MAP2* and *DCX* transcripts within and between control and PWS iN clones, suggesting similar rates of transduction, induction, and differentiation efficiency (Figure 3A). Similarly, differentiated neuron markers βIII tubulin (TUBB3), MAP2, neurofilament-L, neurofilament-H, and neuron-specific enolase (NSE) were detected in both control and PWS iN (Supplemental Figure 3C). Importantly, we detected a >15-fold increase in *MAGEL2* transcript level in control iN compared with control undifferentiated iPSC and PWS iN (Figure 3B). Next, we used this PWS disease model to investigate alterations to SG proteins. Indeed, quantitative Western blot analysis showed significant reduction of PCSK1, PCSK2, CHGB, and CPE levels in PWS iN compared with control iN (Figure 3, C and D). In accordance to our findings in *Magel2^pΔ/m+^* mice, we observed consistent reduction of SG proteins in PWS iN throughout the entirety of differentiation in comparison with isogenic control iN (Figure 3E). These findings suggest that the abundance of SG proteins is decreased in a human neuronal model of PWS.

### PWS patient neurons derived from DPSCs display impaired endosomal protein trafficking.

To expand our findings directly to a patient-derived PWS neuronal model, we collected DPSCs from PWS (7 deletion and 7 UPD cases) and age-matched control (12) individuals (Supplemental Table 3). DPSCs can be easily acquired from naturally exfoliated teeth (baby teeth) and grown in culture (41). DPSCs are neural crest–derived stem cells that reside in the dentin-pulp complex, and unlike iPSCs, they require no reprogramming. Moreover, DPSCs can be differentiated into various human tissues (42), including cortex-like neurons (43, 44). Therefore, using differentiated DPSCs is a unique model to investigate the role of MAGEL2 in PWS, as they generate patient-derived, disease-relevant neurons from multiple unrelated individuals and capture the heterogeneous genetic backgrounds of actual PWS patients.

**Figure 4:**
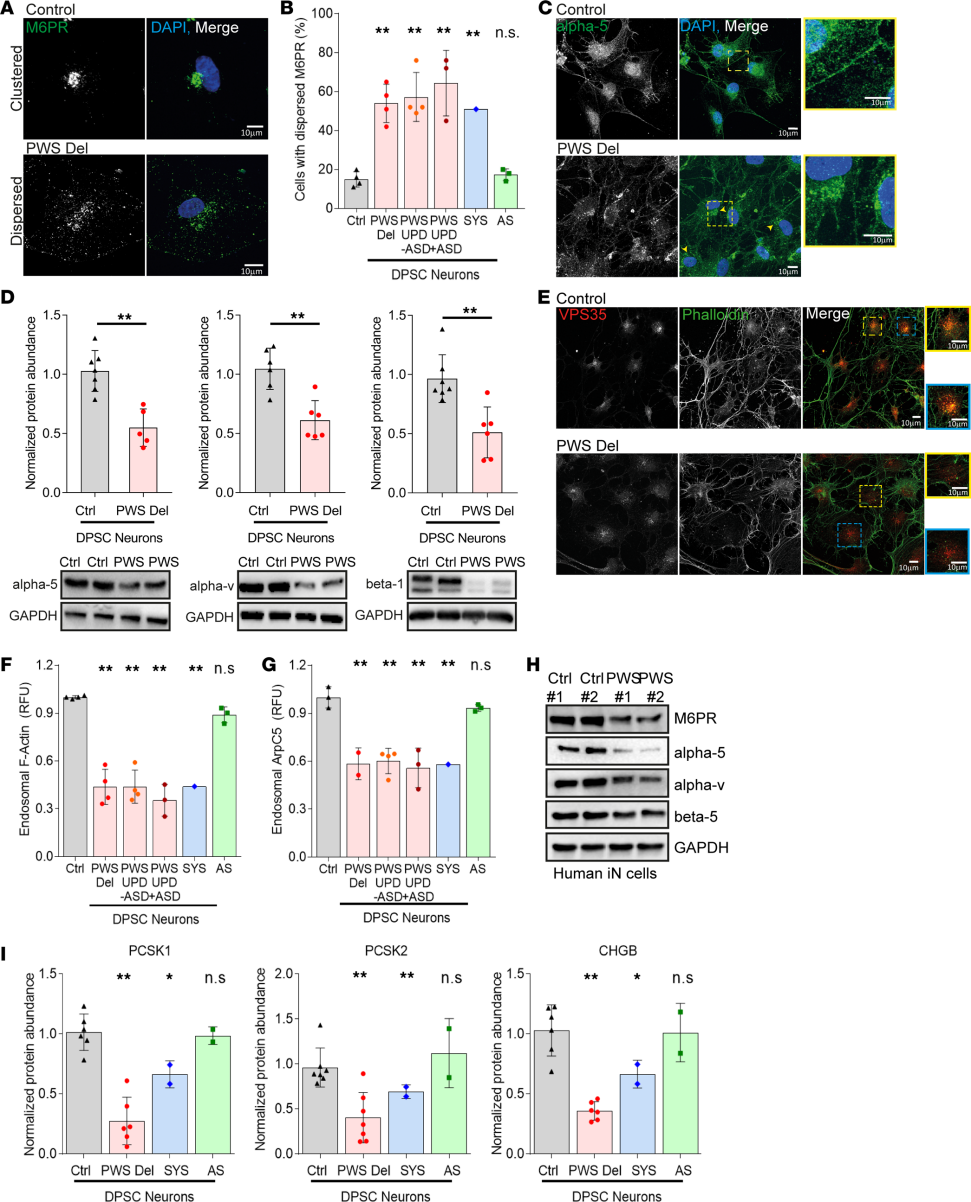
DPSC-derived neurons from PWS patients have impaired endosome-mediated protein trafficking and decreased SG protein abundance. (**A**) Representative images of immunofluorescence staining of M6PR in DPSC-derived neurons. Proper M6PR trafficking shows clustered juxtanuclear localization. (**B**) Proportion of cells with impaired M6PR trafficking is significantly increased in PWS and SYS DPSC-derived neurons. Each data point represents 1 individual, plotted as mean ± SD; more than 75 cells/data point, analyzed by 1-way ANOVA. (**C**) Representative images of immunofluorescence staining of integrin α5 in DPSC-derived neurons. Yellow arrows indicate increased intracellular (endosomal) pools. (**D**) Quantification of Western blot analysis showed reduced expression of integrin α5, αV, and β1 in PWS deletion DPSC-derived neurons. Each target protein is first normalized to GAPDH, then normalized to averaged control. Each data point represents 1 individual (*n* = 6), plotted as mean ± SD and analyzed by unpaired, 2-tailed *t* test. (**E**) Representative images of immunofluorescence staining of phalloidin on VPS35-marked endosomes in DPSC-derived neurons. (**F**) Fluorescence intensity of F-actin on VPS35-marked endosomes is significantly decreased in PWS and SYS DPSC-derived neurons. Each data point represents 1 individual, plotted as mean ± SD; more than 75 cells/data point, analyzed by 1-way ANOVA. (**G**) Fluorescence intensity of ArpC5 on VPS35-marked endosomes is significantly decreased in PWS and SYS DPSC-derived neurons. Each data point represents 1 individual, plotted as mean ± SD; more than 75 cells/data point, analyzed by 1-way ANOVA. (**H**) Western blot analysis of M6PR, α5, αV, and β5 between control and PWS iN. GAPDH served as loading control. (**I**) Quantification of Western blot analysis showed reduced expression of PCSK1, PCSK2, and CHGB in PWS deletion and SYS DPSC-derived neurons. Each target protein is first normalized to GAPDH, then normalized to averaged control. Each data point represents 1 individual, plotted as mean ± SD and analyzed by unpaired, 2-tailed *t* test. *P < 0.05 and **P < 0.01. Scale bars: 10 μm.

Because MAGEL2 cellular functions have previously been investigated only in cancer cell models, we first examined the role of MAGEL2 in endosome-mediated protein trafficking in DPSC-derived neurons to validate this PWS model system. To start, we examined endosome-mediated retrograde transport of cation-dependent mannose-6-phosphate receptor (M6PR) that has previously been shown to be dependent on MAGEL2 in other cell types (15, 16). Using a previously described assay for quantification of M6PR trafficking (15, 16), where proper trafficking results in recycling back to the TGN that is observed as compact juxtanuclear localization, whereas impairment of M6PR trafficking results in endosomal accumulation throughout the cellular volume (Figure 4A), examination of PWS DPSC-derived neurons showed a significant increase in mistrafficked M6PR in PWS DPSC-neurons (Figure 4B). Interestingly, we noted similar levels of impaired trafficking between different PWS genotypes, paternal deletion or maternal UPD, and independent of ASD diagnosis (Figure 4B). We also examined DPSC-derived neurons from an SYS patient with c.1996dupC *MAGEL2* mutation, which also showed impaired M6PR trafficking (Figure 4B). In addition to using age-matched subjects as controls, we examined M6PR trafficking in neurons derived from DPSCs from Angelman syndrome (AS) patients, a phenotypically distinct paternally imprinted neurogenetic disorder caused by maternal deletions of the same 15q11.2-q13 chromosomal region (45). M6PR trafficking was not affected in AS patient DPSC-neurons, highlighting the specificity of this phenotype to PWS and SYS, where imprinted expression of MAGEL2 is disrupted (Figure 4B). Consistent with MAGEL2 being primarily restricted to expression in neurons (16, 32), M6PR was trafficked properly in PWS-derived undifferentiated DPSCs and DPSC-derived adipocytes that do not express MAGEL2 (16) (Supplemental Figure 4A). In addition to M6PR, integrins are similarly recycled in a MAGEL2-dependent fashion from endosomes to the plasma membrane (15, 16). Consistent with M6PR, we noted a reduction of integrin α5 accumulation at the plasma membrane and accumulation in cytoplasmic vesicles of PWS DPSC-derived neurons in comparison with control DPSC-derived neurons (Figure 4C). Concurrently, we quantified a significant decrease in the total abundance of integrins α5, αV, and β1 in PWS DPSC-derived neurons by Western blot analysis (Figure 4D), supporting that aberrant cell surface recycling of integrins results in their degradation. Again, aberrant degradation of integrins was not observed in undifferentiated PWS DPSCs (Supplemental Figure 4B). Thus, PWS patient–derived DPSC-neurons have impaired endosomal recycling pathways.

Previously we showed loss of MAGEL2 resulted in insufficient F-actin nucleation on endosomes that affected downstream protein trafficking (15, 16). Therefore, we examined F-actin and ArpC5 accumulation on endosomes in PWS DPSC-derived neurons. Indeed, we noted a significant reduction of both F-actin (Figure 4, E and F) and ArpC5 (Figure 4G) fluorescence signals on VPS35-marked endosomes in PWS and SYS DPSC-derived neurons. Consistently, these MAGEL2-associated defects were not observed in undifferentiated DPSCs, adipocytes derived from the same DPSC lines, or DPSC-neurons from patients with AS (Figure 4, F and G; Supplemental Figure 4, C and D). We also quantified a significant decrease in the total abundance of integrins and M6PR in PWS iN by Western blot analysis (Figure 4H). These findings demonstrate that DPSC-derived neurons from PWS patients and PWS iN recapitulate defects in endosome trafficking observed upon depletion of MAGEL2 in other cellular models.

Given the establishment of DPSC-derived neurons as a model system for the study of PWS, we followed up on our findings in *Magel2^pΔ/m+^* mice and PWS iN to determine whether SG protein abundances are altered in these cells. Western blot analysis showed nearly 50% reduction in PCSK1, PCSK2, and CHGB protein levels in PWS DPSC-neurons compared with DPSC-neurons derived from age-matched control individuals or DPSC-neurons derived from AS individuals (Figure 4I). Consistent with this effect being due to loss of MAGEL2, SG protein levels were also reduced in SYS patient DPSC-derived neurons that have a mutation in MAGEL2 and not loss of other PWS locus genes (Figure 4I). Together, these findings suggest that the SG pathway is altered in PWS and SYS patient–derived neurons.

### WASH-mediated protein trafficking prevents lysosomal degradation of SG proteins.

**Figure 5:**
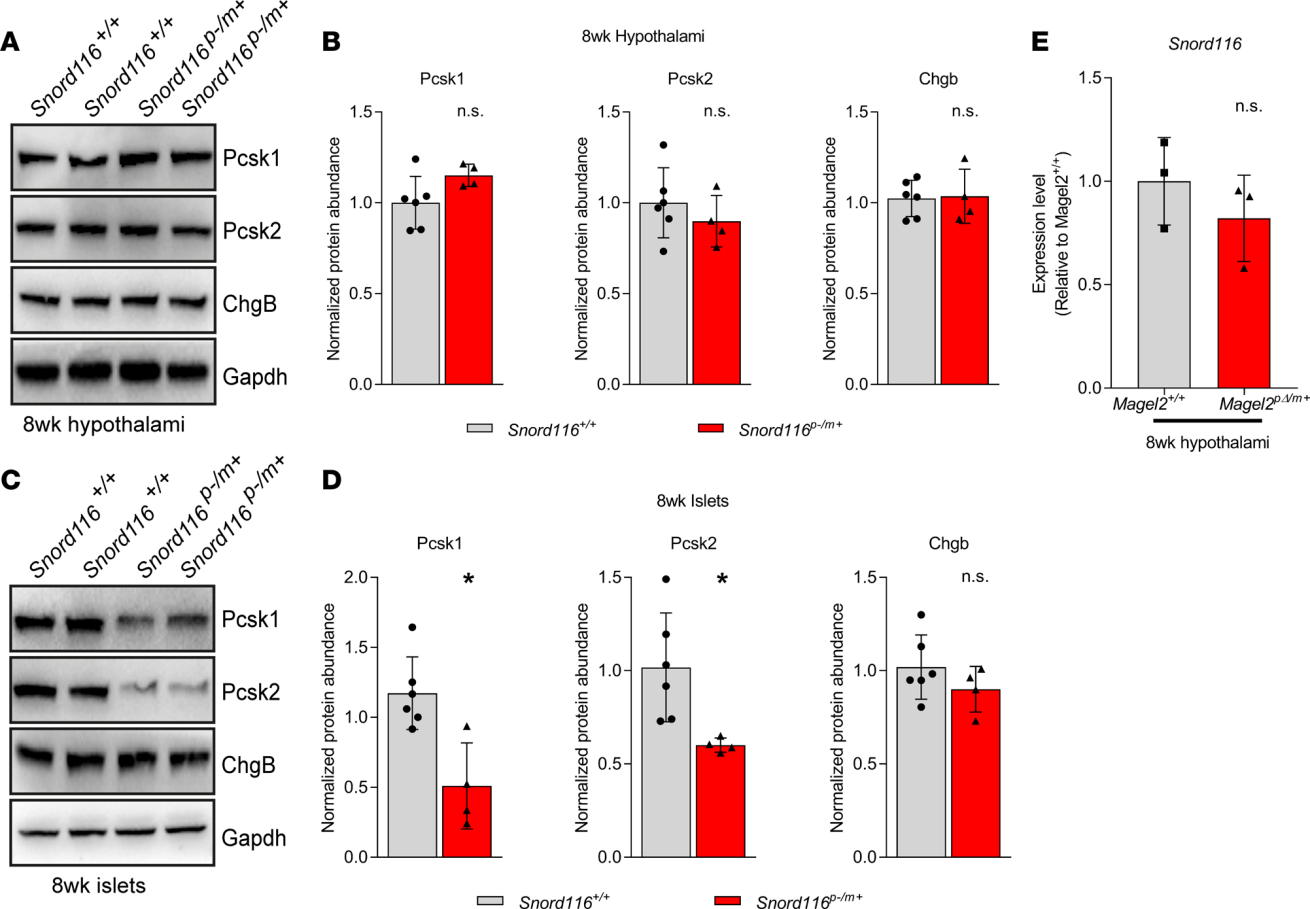
SG protein levels are reduced in pancreatic islets, but not hypothalamic tissue, from *Snord116^p–/m+^* mice. (**A** and **B**) Western blot analysis of Pcsk1, Pcsk2, and Chgb in 8-week-old *Snord116^p–/m+^* mouse hypothalamic tissue. Gapdh served as a normalization loading control. Each data point represents 1 animal, plotted as mean ± SD and analyzed by unpaired, 2-tailed *t* test. (*n* > 4 per genotype.) (**C** and **D**) Western blot analysis of Pcsk1, Pcsk2, and Chgb in 8-week-old *Snord116^p–/m+^* mice islets. Gapdh served as normalization loading control. Each data point represents 1 animal, plotted as mean ± SD and analyzed by unpaired, 2-tailed *t* test, *P > 0.05. (*n* > 4 per genotype.) (**E**) *Snord116* transcript levels are comparable between 8-week-old *Magel2*^p*Δ*/m+^** and *Magel2^+/+^* mouse hypothalamuses. Each data point represents 1 animal (*n* = 3 per genotype), plotted as mean ± SD and analyzed by unpaired, 2-tailed *t* test.

Next, we sought to determine the molecular mechanism by which SG protein abundance is decreased by loss of MAGEL2 in PWS. As PWS is a multigene disease, loss of other genes in the locus may also play a role in SG regulation. Specifically, it is unclear whether deletion of the small nucleolar RNA cluster *Snord116* in mice downregulates Pcsk1 levels in the hypothalamus (46, 47). Burnett et al. reported a trend toward a decrease in *Pcsk1* mRNA in the *Snord116^p–/m+^* hypothalamus, although this was only observed during fasting and did not reach statistical significance (46). A follow-up study by Polex-Wolf et al. found no decrease in *Pcsk1* transcript during both fasting and refeeding in the *Snord116^p–/m+^* hypothalamus (47). To dissect whether *Magel2* alone or along with *Snord116* regulates SG protein abundance in the hypothalamus, we measured protein expression of Pcsk1, Pcsk2, and Chgb in hypothalamic tissues from *Snord116* paternal knockout (*Snord116^p–/m+^*) mice. Abundance of these proteins was unaltered in *Snord116^p–/m+^* mice compared with littermate controls (Figure 5, A and B). However, consistent with previous results (46) we noted a nearly 50% reduction in Pcsk1 and Pcsk2 protein levels in pancreatic islets from *Snord116^p–/m+^* mice (Figure 5, C and D). Importantly, *Snord116* levels were not altered in the hypothalamus of *Magel2^pΔ/m+^* mice (Supplemental Figure 5E). These findings suggest that Magel2, but not Snord116, plays a major role in regulating SG protein abundance in the mouse hypothalamus.

**Figure 6:**
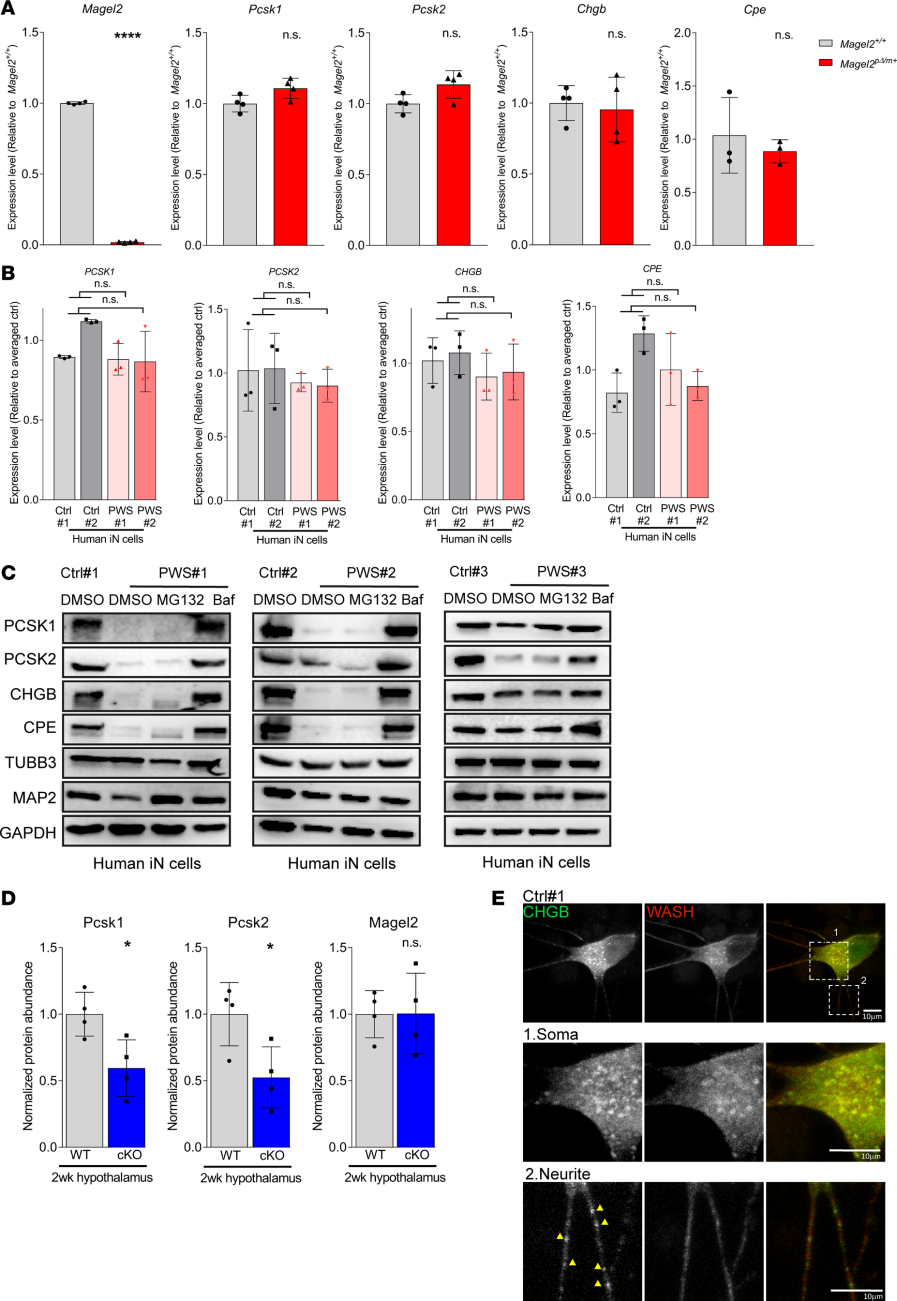
WASH-mediated protein trafficking prevents lysosomal degradation of SG proteins. (**A**) Transcript levels of *Pcsk1*, *Pcsk2*, *Chgb*, and *Cpe* in 8-week-old *Magel2*^p*Δ*/m+^** and *Magel2^+/+^* mouse hypothalamuses, *n* > 3 per genotype. Each data point represents 1 animal, plotted as mean ± SD and analyzed by unpaired, 2-tailed *t* test, ****P < 0.0001. (**B**) Transcript levels of *PCSK1*, *PCSK2*, *CHGB*, and *CPE* between control and PWS iN at 14 days postinduction. Each data point represents 1 induction experiment (*n* = 3), plotted as mean ± SD and analyzed by 1-way ANOVA. (**C**) Western blot analysis shows rescued expression of PCSK1, PCSK2, CHGB, and CPE in PWS iN at 14 days postinduction following inhibition of lysosomes by Baf (1 μM, 7 hours) but not inhibition of proteasomes by MG132 (10 μM, 7 hours). DMSO served as vehicle control. GAPDH served as loading control. (**D**) Quantification of Western blot analysis confirmed reduced expression of Pcsk1 and Pcsk2 in 2-week-old *Washc1* conditional knockout (cKO) mouse hypothalamuses, *n* = 4 per genotype. Gapdh served as loading control. Each data point represents 1 animal, plotted as mean ± SD and analyzed by unpaired, 2-tailed *t* test, *P < 0.05. (**E**) Representative images of immunofluorescence staining with CHGB and WASH at both soma and neurite in control iN. Scale bars indicate 10 μm.

To dissect out how *Magel2* contributes to loss of SG protein abundance in the hypothalamus, we measured transcript levels of *Pcsk1*, *Pcsk2*, *Chgb*, and *Cpe* by qRT-PCR. We observed no significant differences in the levels of these transcripts between WT and *Magel2^pΔ/m+^* mice at 2, 8, or 20 weeks (Figure 6A and Supplemental Figure 5, A and B). Furthermore, we observed no difference in the transcript levels of these genes in PWS iN as compared with isogenic controls throughout the entirety of neuronal induction (Figure 6B and Supplemental Figure 5C). These findings suggest that loss of SG protein abundance is likely attributable to posttranscriptional differences, such as alterations in protein stability. To test this possibility, we treated PWS iN cultures with MG132 to inhibit proteasome-mediated protein degradation or bafilomycin A1 (Baf) to inhibit lysosome-mediated protein degradation. Western blot analysis revealed nearly complete rescue of PCSK1, PCSK2, CHGB, and CPE abundance with Baf treatment but not with MG132 (Figure 6C).

To confirm our findings, we generated an additional isogenic pair of control (Ctrl 3) and type I PWS deletion (PWS 3) iPSC lines from a genetically distinct individual (male 8330 cells) by CRISPR/Cas9 editing. Consistent with our previous findings, the abundance of SG proteins was substantially decreased in PWS 3 iN compared with Ctrl 3 iN (Figure 6C). Importantly, this defect was due to lysosomal degradation, as Baf, but not MG132, rescued levels of SG proteins in PWS 3 iN (Figure 6C). Consistent with the aberrant lysosomal degradation of SG proteins in PWS iN, we observed colocalization between CHGB and LAMP1 lysosomal protein in PWS iN (Supplemental Figure 5D). Furthermore, the transcript levels of SG genes were not significantly different in PWS 3 iN compared with Ctrl 3 iN (Supplemental Figure 5E).

Given our findings that SG proteins underwent lysosomal degradation in PWS iN and a recent report that endosome-derived retrograde trafficking promotes SG maturation (48), we hypothesized that dysregulation of WASH-mediated protein trafficking upon loss of MAGEL2 may play a role in regulating SG protein levels. To test this, we crossed previously described (49) *Washc1*^fl/fl^ mice with *Nestin-cre* mice to selectively knock out *Washc1* in neuronal lineages. Although these animals are not viable long term, analysis of hypothalamic tissues at 2 weeks of age revealed significant reduction in Pcsk1 and Pcsk2 protein levels in knockout animals compared with WT littermate controls (Figure 5D and Supplemental Figure 6A), although Chgb was not significantly decreased (Supplemental Figure 6A). Consistent with a role of WASH and retromer recycling pathways in SG biogenesis and/or maturation, we observed colocalization of both CHGB and CHGA with SHRC components (WASH and FAM21) and retromer protein VPS35 within the soma of human iN but not in neurite processes where mature SGs are stored (Figure 5E and Supplemental Figure 6, B–E). These observations suggest that WASH-mediated endosomal protein trafficking is required for SG maturation and when dysregulated leads to aberrant lysosomal degradation of SG components.

### SG abundance is reduced in the absence of MAGEL2.

**Figure 7:**
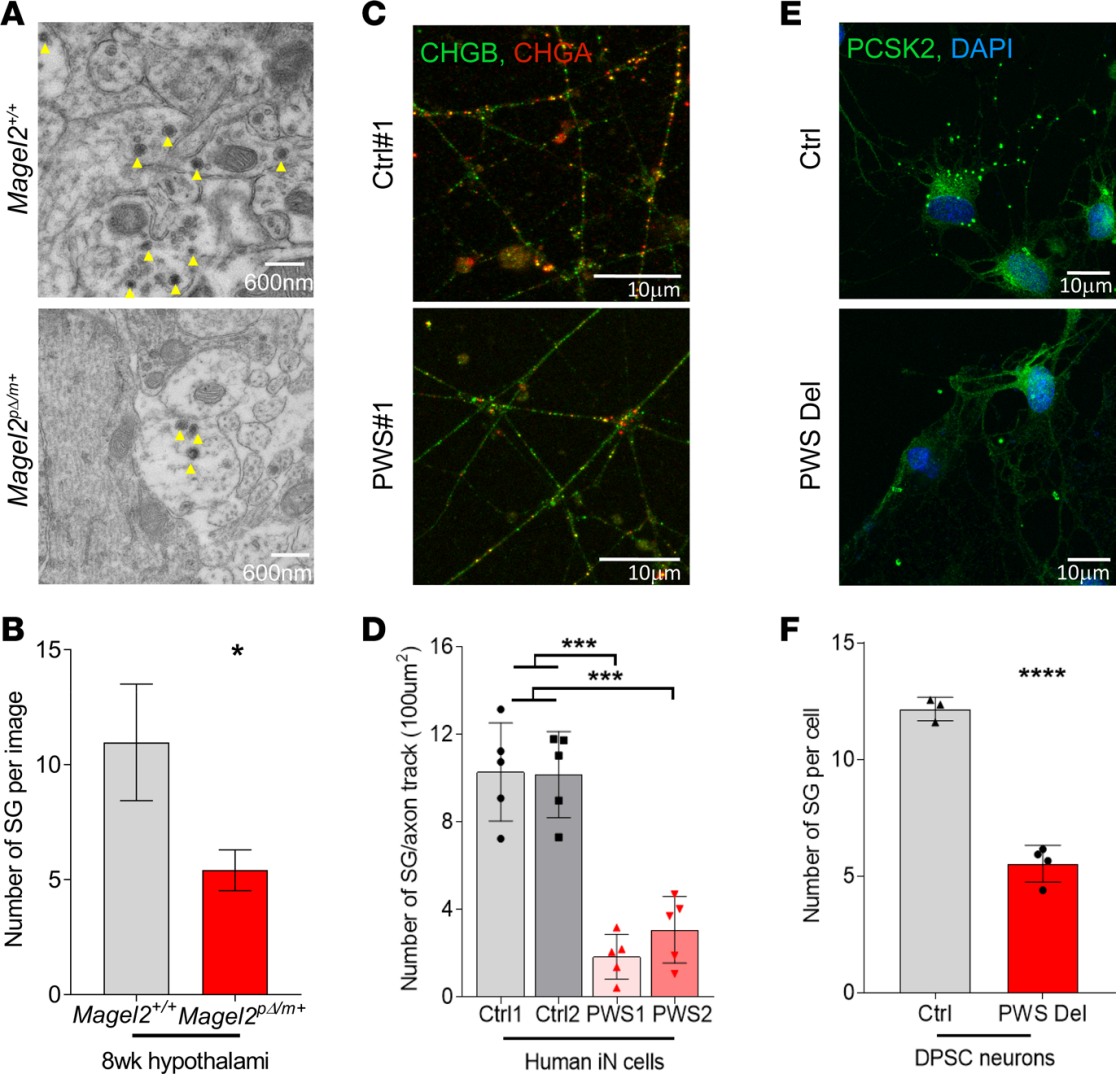
SG biogenesis is impaired *Magel2^pΔ/m+^* mice, human iN with PWS deletion, and DPSC-derived neurons in PWS patients. (**A** and **B**) Representative images (**A**) and quantitation (**B**) of SG (yellow arrows) by electron microscopy of 8-week-old *Magel2^+/+^* and *Magel2*^p*Δ*/m+^** *^+^* mouse medial eminence region of hypothalamus, *n* = 2 per genotype; >30 images were analyzed. Data shown as mean ± SD and analyzed by unpaired, 2-tailed *t* test; *P < 0.05. (**C** and **D**) Representative images (**C**) and quantitation (**D**) of immunofluorescence staining with CHGB and CHGA in control and PWS iN. Each data point represents 1 induction experiment (*n* = 5), plotted as mean ± SD and analyzed by 1-way ANOVA. (**E** and **F**) Representative images (**E**) and quantitation (**F**) of immunofluorescence staining with PCSK2 and DAPI in control and PWS deletion DPSC-derived neurons. Each data point represents 1 individual, plotted as mean ± SD and analyzed by unpaired, 2-tailed *t* test. *P < 0.05, ***P < 0.001, and ****P < 0.001.

Next, we determined whether reductions in SG proteins resulted in alterations in the number of SGs in our model systems. We used electron microscopy to quantify the number of SGs in hypothalamic tissue collected from 8-week-old *Magel2^pΔ/m+^* mice and control *Magel2^+/+^* littermates. SGs are readily identified by electron microscopy given their unique structure of a highly dense core surrounded by membrane (36, 50). Within the hypothalamus, we focused on the median eminence region that interfaces the hypothalamus with the peripheral endocrine system of the pituitary. Most of the SGs were uniform in size, with diameters of 100–150 nm in both *Magel2^pΔ/m+^* and control *Magel2^+/+^* mice (Figure 7A). However, we noted a significant reduction in the number of SGs in *Magel2^pΔ/m+^* mice compared with the *Magel2^+/+^* littermate controls (Figure 7B). To determine if these defects were apparent in human PWS neuronal models, we performed automated analysis of human iN stained for CHGA and CHGB SG proteins and noted a dramatic reduction in SGs in PWS deletion iN in comparison with isogenic control iN (Figure 7, C and D). Last, we quantified PCSK2-marked SGs in DPSC-derived neurons from PWS patients and consistently observed decreased number of SGs per cell compared with DPSC-derived neurons from healthy control subjects (Figure 7, E and F). Thus, both SG protein abundance and SG numbers are reduced upon loss of MAGEL2.

### Neuropeptide production is impaired in Magel2^pΔ/m+^ mice and human PWS iN.

**Figure 8:**
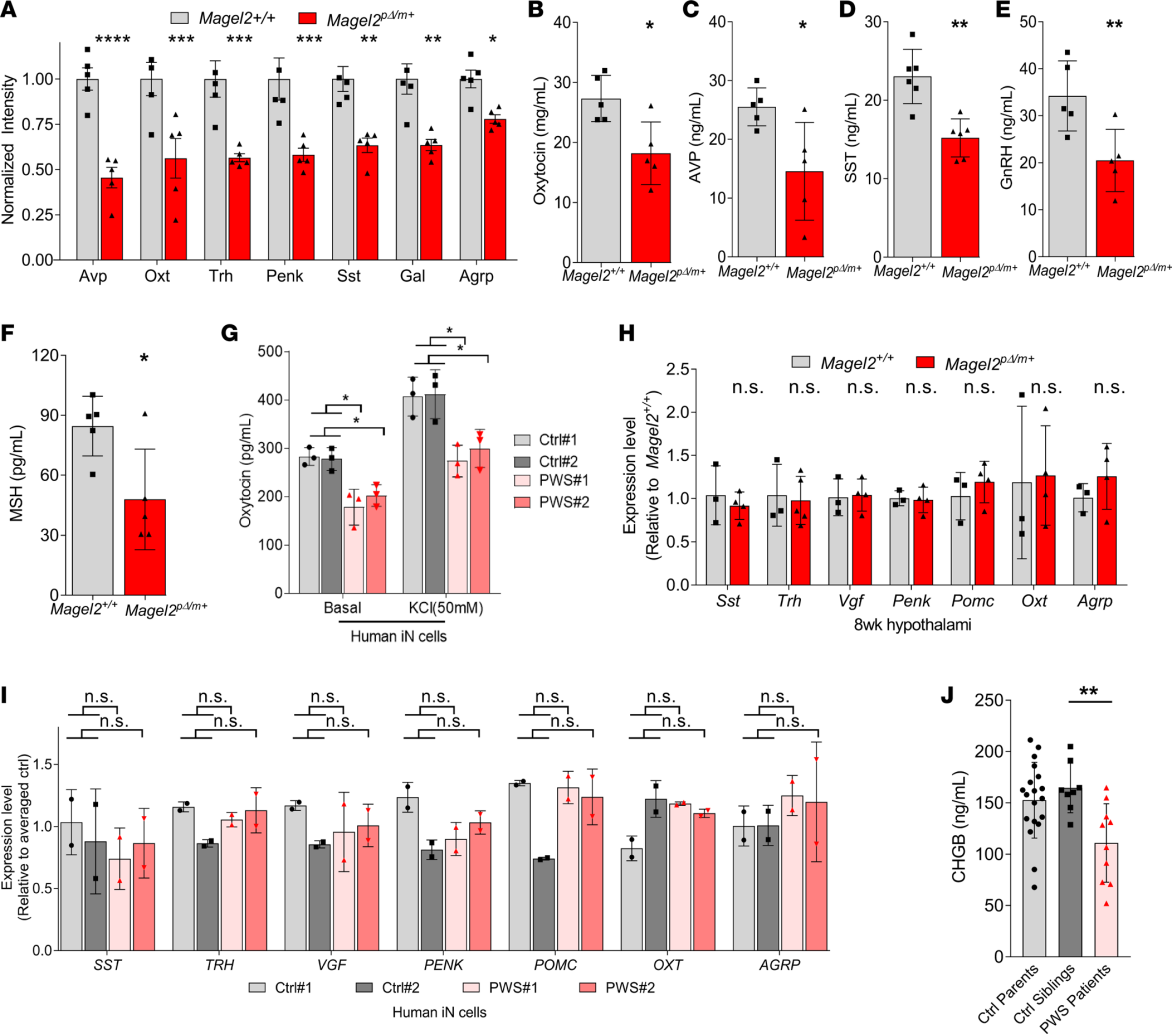
Neuropeptide production and release are impaired in *Magel2^pΔ/m+^* mice and human PWS iN. (**A**) Peptide abundance of indicated neuropeptides in 8-week-old *Magel2^+/+^* and *Magel2*^p*Δ*/m+^** mouse hypothalamuses. Each data point represents 1 animal, plotted as mean ± SD (*n* = 5 per genotype) and indicated *P* values as analyzed by unpaired, 2-tailed *t* test with Bonferroni’s correction. (**B**–**F**) Reduced levels of plasma OXT (**B**), AVP (**C**), SST (**D**), GnRH (**E**), and α-MSH (**F**) in 14-week-old *Magel2*^p*Δ*/m+^** mice, measured by ELISA. Each data point represents 1 animal, plotted as mean ± SD (*n* > 5 per genotype), and analyzed by unpaired, 2-tailed *t* test. (**G**) ELISA analysis of oxytocin levels in human iN media, at basal level and after KCl (50 mM, 30 minutes) stimulation. Each data point represents 1 induction experiment, plotted as mean ± SD (*n* = 3), and analyzed by 1-way ANOVA. (**H**) Transcript levels of various neuropeptides between 8-week-old *Magel2*^p*Δ*/m+^** and *Magel2^+/+^* mouse hypothalamuses. Each data point represents 1 animal, plotted as mean ± SD (*n* = 3 per genotype) and analyzed by unpaired, 2-tailed *t* test. (**I**) Transcript levels of various neuropeptides between control and PWS iN at 14 days postinduction. Each data point represents 1 induction experiment (*n* = 2), plotted as mean ± SD and analyzed by 1-way ANOVA. (**J**) Reduced levels of plasma CHGB in PWS patients after 12 hours of fasting, measured by ELISA. Each data point is a unique individual, plotted as mean ± SD and analyzed by Mann-Whitney *U* test. *P < 0.05, ***P < 0.001, and ****P < 0.001.

Various hormone deficiencies are universal features of PWS pathology. For example, oxytocin is an anorexigenic hormone important for body weight regulation that promotes bonding and social behaviors and is decreased in patients with PWS (28, 51, 52). Reduction in oxytocin also contributes to impaired suckling behavior in infants and has been implicated in ASD (53). Consistent with these associations, previous analysis of hypothalamic tissues collected from *Magel2^pΔ/m+^* mice revealed a significant 36% reduction of amidated mature oxytocin compared with WT mice (54). In accordance, we noted an approximately 50% reduction of oxytocin gene products in *Magel2^pΔ/m+^* mice by quantitative TMT proteomics (Figure 8A), along with several other PWS-relevant neuropeptides, such as AVP that in addition to endocrine functions affects behaviors related social interactions and SST that regulates growth hormone, insulin, glucagon, and other endocrine secretions (51). Given that our proteomic analysis cannot distinguish between prohormones and cleaved bioactive hormones, we performed ELISAs to examine bioactive hormones in sera collected from *Magel2^pΔ/m+^* mice. We observed reduced levels of the PCSK1/2 substrates OXT, AVP, SST, gonadotropin-releasing hormone (GnRH), and melanocyte-stimulating hormone (MSH) in *Magel2^pΔ/m+^* mice compared with *Magel2^+/+^* littermates (Figure 8, B–F).

To determine whether similar defects were present in PWS iN, we stimulated 14-day-old iN with KCl for 1 hour and measured the concentration of oxytocin in the medium by ELISA. KCl stimulated the secretion of oxytocin in both control and PWS iN by nearly 30% (Figure 8G). Importantly, in both unstimulated and KCl-stimulated conditions, we detected significantly more oxytocin released into culture media in control iN than PWS iN (Figure 8G). The decreased abundance of oxytocin and other neuropeptides was not attributable to transcript-level differences in mouse hypothalamic tissue or iN (Figure 8, H and I; and Supplemental Figure 7, A, B, and D). Last, we determined whether secretion through the SG pathway is altered in patients with PWS. CHGB is secreted along with bioactive hormones upon stimulation of the regulated secretory pathway and serves as a marker for SG production and content release (50). ELISA analysis showed reduced levels of plasma CHGB in PWS patients (Supplemental Table 4) compared with unaffected siblings and healthy controls (Figure 8J). Collectively, these findings suggest that loss of SG abundance and downstream neuropeptide release are hallmark features of PWS.

## Discussion

PWS is a contiguous gene disorder characterized by the loss of paternal expression for several genes at the chromosome 15q11-q13 locus. The molecular basis for disease phenotypes and the underlying gene(s) responsible within the PWS locus have been unclear. The clinical phenotypes of PWS implicate a disturbance of neuroendocrine pathways, including the hypothalamus (55–57). Here, we identified MAGEL2 as an important regulator of SG biogenesis and neuropeptide production in the hypothalamus. In addition to SYS, truncating mutations of *MAGEL2* cause arthrogryposis multiplex congenita (OMIM #208100) (58) and Chitayat-Hall syndrome (OMIM #208080) (59) that both display global developmental delay and growth hormone deficiency, similar to PWS and SYS. Based on the known cellular role of MAGEL2 in regulating endosomal protein trafficking and recycling (14–16, 22), we hypothesized that neuroendocrine proteins may be missorted and degraded in the hypothalamus when MAGEL2 is lost. We applied an unbiased proteomics approach to identify proteins that are downregulated in *Magel2^pΔ/m+^* mice and identified a small group of proteins significantly decreased. Gene ontology analysis revealed these downregulated proteins were involved in the regulated SG pathway for neuropeptide processing, trafficking, and secretion (Figure 2B and Supplemental Table 1). We developed and used PWS neuronal culture model systems and observed a reduction in many SG components, including loss of granins, that enhance prohormone aggregation during SG biogenesis in the TGN and are quantitatively the major constituents of SG (50, 60). Previous reports have shown that SG abundance is positively correlated with the expression levels of CHGB and CHGA (50, 60). Accordingly, we observed significant reduction in SG numbers in neuronal terminals in the median eminence region of the hypothalamus of *Magel2^pΔ/m+^* mice. Similar findings were observed in cortex-like neuronal models derived from PWS iPSCs and DPSCs (Figure 7).

Mechanistically, we uncovered that upon MAGEL2 loss, SG proteins were reduced because of lysosomal degradation and depended on the endosomal F-actin nucleator WASH (Figure 6), suggesting that MAGEL2-dependent regulation of WASH (15) is critical for SG biogenesis and maturation. Using PWS DPSC-derived neurons, we observed severe defects in the retromer and WASH-dependent endosomal recycling pathways (Figure 4). Indeed, several components of SGs are known cargos of the retromer/WASH pathway, including sortilin, furin, V-ATPase, and M6PR (19, 20). Interestingly, these components are only present in immature SGs, and it has been suggested that they are recycled back to the TGN through retrograde transport (35). In support of this, we observed colocalization between granins and the retromer complex component VPS35. Additionally, endosomal membrane remodeling by localized F-actin is an important aspect in protein trafficking and SG maturation (25). Thus, localized F-actin may be important for SG budding of immature SGs from the TGN or their subsequent maturation. Intriguingly, a recent genetic screen in the *Drosophila* larval salivary gland identified a large number of endosomal trafficking genes required for proper SG maturation (48). Thus, detailed mechanistic studies are vital to understand how endosomal recycling pathways and F-actin impinge upon SG biogenesis and neuropeptide production.

We observed a significant decrease in neuropeptide processing enzymes PCSK1, PCSK2, and CPE in both human and mouse PWS models (Figure 2, Figure 3, and Figure 4). Consequently, we detected reduced levels of secreted components of SGs, including various bioactive hormones in *Magel2^pΔ/m+^* mice (Figure 8), similar to those reported in *Pcsk1*-deficient mice (46). Consistent with our findings, previous studies showed reduced hormone processing in *Magel2*-deficient mice, including lower circulating mature orexin, but increased unprocessed prepro-orexin (4), decreased oxytocin (10, 54), and reduced release of growth hormone (12). Additionally, a PWS deletion mouse model showed reduced insulin secretion (61). These findings extend to humans, where patients with PWS have been reported to have decreased vasopressin and oxytocin (62, 63). Defects in neuropeptide processing and release are consistent with hormonal imbalance in PWS that may contribute to hyperphagic obesity, hypogonadism, growth hormone deficiency, hyperghrelinemia, and hypoinsulinemia (56, 57, 64). Consistent with reduced neuropeptide production being important in PWS and Magel2 disease models, a single postnatal injection of oxytocin rescues the lethal feeding behavior in Magel2-deficient mice (10).

A previous study using iPSC-derived neurons from PWS patients reported decreased PCSK1 protein abundance along with hormone processing defects (46). Furthermore, decreased PCSK2 protein levels have been observed in the PVN of the hypothalamus in PWS patient postmortem tissue (62). These studies are consistent with our findings that MAGEL2 and possibly other genes within the PWS locus play an important role in regulating PCSK1/2 levels. Burnett et al. and Polex-Wolf et al. agree that *Pcsk1* transcripts are not significantly reduced in paternal deletion *Snord116* mouse hypothalamic tissues (46, 47), whereas Burnett et al. showed downregulation of *Pcsk1* and *Pcsk2* transcript and protein levels in pancreatic islets from *Snord116^p–/m+^* mice (46). In our study, we confirmed these findings and noted no significant changes to *Snord116* transcript levels in *Magel2^pΔ/m+^* mouse hypothalamuses (Figure 5E). This suggests that Magel2 regulates SG abundance independent of *Snord116*. Thus, in mice, it appears that deficiency of *Magel2* mediates downregulation of Pcsk1 protein and SG components in the hypothalamus, while primary generalized congenital genetic deficiency of *Snord116* causes downregulation of *Pcsk1* transcript and protein in peripheral tissues, such as the endocrine pancreas and stomach.

It is important to note that a discrepancy does exist between the human data reported here and those of Burnett et al. Specifically, iPSC-derived neurons generated by chemical induction from a single individual with a paternal microdeletion encompassing *SNORD109A*, *SNORD116*, and *IPW*, as well as 3 individuals with PWS large deletion genotypes, showed reduced PCSK1 protein abundance by downregulation at the transcript level (46). We find that MAGEL2 regulates the abundance of PCSK1 (and other SG proteins) in iNeurons derived by NGN2 expression through control of lysosomal degradation pathways, not alterations in transcript levels of *PCSK1*. It is likely that differences in neuronal differentiation methodology (NGN2 induced versus chemically induced) or cell culture conditions/timing may account for the discrepancy in *PCSK1* transcript levels found in these PWS large deletion stem cell–derived neurons. Differences between human *SNORD116* and mouse *Snord116* may underlie differences in apparent PCSK1 regulation in neuronal tissues. Ultimately further studies using systematic, isogenic deletion of each gene in the PWS region will be needed to fully elucidate specific individual contributions of human *SNORD116* and *MAGEL2* in the regulation of PCSK1.

Collectively, our findings suggest that multiple genes in the PWS locus, including *MAGEL2* and *SNORD116*, functionally converge in their regulation of PCSK1 to support production of SG and neuropeptide processing and release in mechanistically distinct ways in different secretory cell types (e.g., hypothalamic and other neurons, pituicytes, stomach, β cells). Loss of paternal MAGEL2 expression results in decreased abundance of SG proteins because of aberrant endosomal protein trafficking and lysosomal degradation; consequently, the abundance of mature SGs within neurons and circulating bioactive hormones are reduced. Our study illustrates the importance of PWS locus gene products in SG and neuropeptide production and handling and suggests that restoration of this cellular pathway may hold potential to alleviate several pathogenic processes in PWS.

## Methods

### Antibodies.

For detection of endogenous Magel2, a rabbit polyclonal antibody was generated with recombinant mouse Magel2 protein (amino acids 970–1284) (Proteintech). Serum was purified against the recombinant Magel2 protein. The following commercial antibodies were used: ArpC5 (Synaptic Systems, 305011), α5 (BD Pharmingen, 555615), α5 (Cell Signaling Technology, 4705), αV (Cell Signaling Technology, 4711), β1 (Cell Signaling Technology, 9699), LAMP1 (Abcam, ab208943), M6PR (Abcam, ab2733), VPS35 (Abcam, ab10099), PCSK1 (Cell Signaling Technology, 11914), PCSK2 (Cell Signaling Technology, 14013), CHGB (MilliporeSigma, HPA008759), CPE (R&D Systems, Bio-Techne, AF3587), MAP2 (Abcam, ab5392), TUBB3 (Cell Signaling Technology, 5568), β-tubulin (MilliporeSigma, T8328), Phalloidin (Invitrogen, Thermo Fisher Scientific, A22287), GAPDH (Cell Signaling Technology, 5174), CHGA (R&D Systems, Bio-Techne, MAB90981), WASH (MilliporeSigma, SAB4200552), FAM21 (MilliporeSigma, ABT79), NSE (BioLegend, 804906), neurofilament-H (BioLegend, 801709), and neurofilament-L (Cell Signaling Technology, 2837). Secondary antibodies were conjugated to HRP (MilliporeSigma) or to Alexa Fluor 488 (Thermo Fisher Scientific, A32723 and A32731), Alexa Fluor 546 (Thermo Fisher Scientific, A11030 and A11035), or Alexa Fluor 647 (Thermo Fisher Scientific, A32733 and A32728). DAPI was purchased from MilliporeSigma (D4592).

### Lentiviral vector production and titration.

Production and titration of lentiviral vectors were performed as previously described (65) and detailed in the Supplemental Methods.

### Western blot analysis.

Whole cell/tissue lysates were obtained by lysing frozen cell pellets in RIPA buffer (Thermo Fisher Scientific) with protease/phosphatase inhibitors (Thermo Fisher Scientific). Cell lysates were sonicated for 15 seconds, while tissue lysates were homogenized for 30 seconds. Lysates were clarified by centrifugation at 16,000*g* for 10 minutes at 4°C, and protein concentration was determined using the BCA assay (Pierce, Thermo Fisher Scientific). Clarified lysates were then mixed with SDS sample buffer, resolved on SDS-PAGE gels (Bio-Rad), transferred to nitrocellulose membranes, and then blocked in TBS-Tween with 5% (*w/v*) milk powder. Membranes were blotted with indicated primary antibodies overnight at 4°C and detected by HRP-conjugated antibodies and enhanced chemiluminescence (GE Healthcare). See supplemental materials for uncut gels.

### Mouse colony breeding, maintenance, and tissue collection.

All animal work was carried out with approval of the IACUC of St. Jude Children’s Research Hospital under protocol 597 and Mayo Clinic IACUC protocol A21314. *Magel2^pΔ/m+^* mice on a C57BL/6J background were ordered from The Jackson Laboratory (stock number 009062). The colony was maintained by mating female *Magel2^p+/m–^* with male *Magel2^+/+^* to produce *Magel2^p+/m–^* and *Magel2^+/+^* mice. Phenotypically mutant mice were generated by mating male *Magel2^pΔ/m+^* and female *Magel2^+/+^* to produce *Magel2^pΔ/m+^* and *Magel2^+/+^* offspring. Briefly, tail snips (1–2 mm) were collected at weaning (~21 days old) and again when animals were euthanized for organ collection. Genomic DNA was prepared by incubating tails in 200 μL of 50 mM NaOH at 95°C for 35 minutes, allowed to cool for 3 minutes, and neutralized by addition of 20 μL 1 M Tris pH 8.0. PCR was performed using KAPA2G Robust Hotstart PCR kit (KAPA Biosystems) following the manufacturer’s protocol. Buffer A, Enhancer, and 1 μL of gDNA were used. Primers used were as follows: common forward, 5′-ATGGCTCCATCAGGAGAAC-3′; WT reverse, 5′-GATGGAAAGACCCTTGAGGT-3′; and mutant reverse, 5′-GGGATAGGTCACGTTGGTGT-3′. The *Washc1*^fl/fl^ mice (49, 66) were crossed with *Nestin-cre* mice (The Jackson Laboratory, stock number 003771) to generate heterozygous offspring (*Washc1*^fl/+^ *Nestin-cre*^+^), which were crossed to generate *Washc1*^+/+^ *Nestin-cre*^+^, *Washc1*^fl/+^ *Nestin-cre*^+^, and *Washc1*^fl/fl^ *Nestin-cre*^+^ mice for experiments. The *Washc1*-floxed allele and *cre* transgene were detected using primers as previously described (49, 66).

Mouse tissue collections were performed at 1400 hours at peak Magel2 expression (67) or at day 18–19 postnatal for *Washc1*/*Nestin-cre* genotypes. Animals were euthanized by isoflurane inhalation, and tissues were collected after cervical dislocation or exsanguination by vena cava.

### Immunohistochemistry.

Whole mice were perfused with 0.1 M sodium phosphate buffer (pH 7.2) containing 4% (*w/v*) paraformaldehyde. The whole brain was paraffin-embedded for maintenance. Deparaffinization was done using a DISCOVERY XT autostainer (Ventana Medical Systems) with MAGEL2 antibody (1:500). All slides were counterstained with hematoxylin. Bright-field images were taken with an upright Eclipse Ni (Nikon) or constructed from digitized images using Aperio ImageScope (Leica Biosystems).

### Quantitative proteomic/mass spectrometry.

Whole tissues for hypothalamus, adrenal gland, pituitary, and brainstem were transferred to Precellys CK14 lysis tubes (Bertin Corp). For liver and white adipose tissue (WAT), approximately 200 μL of pulverized tissues were used. Cold lysis buffer containing 90% (*v/v*) methanol, 9% (*v/v*) water, and 1% (*v/v*) acetic acid was added to hypothalamus (600 μL), pituitary and adrenal gland (500 μL), liver (200 μL), WAT (200 μL), and brainstem (250 μL). The protein pellet was dissolved in 8 M urea, with 100 μM triethylammonium bicarbonate (TEAB) pH 8.5, then reduced with Tris (2-carboxyethyl) phosphine (TCEP) and alkylated with chloroacetamide.

Protein concentration was measured by BCA assay (Pierce, Thermo Fisher Scientific), and 50 μg of protein was purified by methanol/chloroform precipitation. Protein pellet was dissolved in 8 M urea, with 100 mM TEAB, then reduced with TCEP and alkylated with chloroacetamide. The dissolved protein was diluted in 2 M urea with 100 mM TEAB and 0.5 μg trypsin (Promega) and placed in a 37°C shaker for 16 hours. Proteins were labeled with 0.2 mg tandem mass tags (Thermo Fisher Scientific, 90309, lot SK259407). Then, 10 μg from each sample was combined for peptide fractionation using High pH Reversed-phase Peptide Fractionation Kit (Pierce, Thermo Fisher Scientific).

Please see Supplemental Methods for a detailed description of mass spectrometry. Peptide/protein identification and quantification were determined using IP2 (Integrated Proteomics Applications). The MS raw data files were converted into MS1, MS2, and MS3 format using RawConverter (68) (version 1.1.0.23) with monoisotopic option. For peptide identification, tandem mass spectra were searched against a database including the UniProt mouse database 1 entry per gene (21,982 entries released December 1, 2019) with common contaminants and reversed sequences using ProLuCID (69). Data were filtered using DTASelect (70). Quantitation was calculated with Census version 2.51 and filtered with an intensity value of 5000 and isobaric purity value of 0.6 (71). The Quantitative@COMPARE feature of IP2 was used to determine statistical significance. Data were visualized using Excel (Microsoft) and TIBCO Spotfire (Spotfire). Gene ontology analysis was performed using GOrilla platform (72).

### Generation and maintenance of iPSC lines.

We used our previously developed Single-guide-CRISPR/Cas-targeting-Of-Repetitive-Elements method (73) to generate type I PWS deletion in isogenic human iPSCs, female MGH2069 (2 clones were analyzed and referred to as 1 and 2), and male 8330 (referred to as clone 3) cells (74). We designed guide RNAs (gRNAs) targeting specific pairs of segmental duplication blocks at the PWS locus such that Cas9-induced double-strand DNA breakage and imperfect repair would result in canonical PWS deletions. The design of gRNAs and the reference sequence is based on genome assembly GRCh38. To generate the canonical PWS deletions, the sequences of gRNAs were as follows: gRNA7559: 5′-ATAGTAGCAAAACGCATACT-3′, chromosome 15: 22,588,838–22,588,857 and 28,683,290–28,683,309; gRNA458: 5′-CTCCCTGCCTAGAAGCTGGT-3′, chromosome 15: 23,198,458–23,198,477 and 28,490,195–28,490,214. To minimize off-target editing and maximize efficiency, we incorporated high-fidelity Cas9 and ribonucleoprotein-based delivery as previously described (75). After isolating single cells using FACS and propagating resultant clonal populations, we performed droplet digital PCR (ddPCR) to assess genomic copy number at 2 genes within the chromosome 15q11-q13 PWS region (*NIPA1* and *HERC2*) and 1 gene just outside the region (*APBA2*) as a control. To confirm the parent of origin, we employed an established methylation-specific ddPCR assay (76) using primers and probes targeting the differentially methylated *SNURF-SNRPN* promoter and exon 1. Finally, we performed array comparative genomic hybridization (array CGH) on each PWS model to evaluate genomic integrity, retaining models with the intended PWS deletions and no off-target copy number variants.

iPSCs were maintained as feeder-free cells in mTeSR 1 medium (STEMCELL Technologies) coated with Matrigel (BD Biosciences). iPSCs were passaged at 70% confluence using ReLeSR (STEMCELL Technologies) with Y-27632 Dihydrochloride (PeproTech) and maintained for 4 passages only. iPSCs were cryopreserved in NutriFreeze D10 Cryopreservation Medium (Biological Industries) with Y-27632 Dihydrochloride.

### Generation of iN.

iPSCs were dissociated into single cells using Accutase (STEMCELL Technologies), and 300,000 iPSCs were seeded into 1 well of a Matrigel-coated 6-well plate in mTeSR 1 medium with Y-27632 Dihydrochloride. The next day, lentiviruses encoding NGN2-GFP (Addgene 79823) and rTTA (Addgene 66810) were added to the medium with 4 μg/mL hexadimethrine bromide (MilliporeSigma). The medium was changed every 24 hours for 2–3 days. When transduced iPSCs reached 70% confluence, 1 μg/mL of doxycycline hyclate (MilliporeSigma) was added to induce NGN2-GFP expression. Transduction efficiency was determined by expression of NGN2-GFP. At day 1 of induction, mTeSR 1 medium was changed to BrainPhys Neuronal Medium (STEMCELL Technologies) containing 1× N2 (Thermo Fisher Scientific), 1× B27 (Thermo Fisher Scientific), 20 ng/mL BDNF (PeproTech), 20 ng/mL GDNF (PeproTech), 500 μg/mL Dibutyryl c-AMP (MilliporeSigma), 200 nM l-ascorbic acid (MilliporeSigma), 1 μg/mL natural mouse laminin (Thermo Fisher Scientific), 1 μg/mL doxycycline hyclate, 1 μg/mL puromycin (Thermo Fisher Scientific), and 1 μM cytosine arabinoside (MilliporeSigma). Transduced iPSCs were maintained in supplemented BrainPhys Neuronal Medium, with doxycycline hyclate removed at day 3 after induction, and half of the medium was changed every 3 days till the assay was performed.

### Immunofluorescence, image acquisition, and image analysis.

Cells were fixed with 4% (*v/v*) paraformaldehyde for 10 minutes at room temperature. Cells were permeabilized with PBS containing 0.25% (*v/v*) Triton X-100. Antibodies were diluted in PBS with 0.1% (*v/v*) Triton X-100 and 3% BSA, with primary antibodies incubated overnight at 4°C. Cells were washed 3 times in PBS containing 0.5% (*v/v*) Tween-20 before and after incubations. Slides were mounted with Aqua-Mount Mounting Medium (VWR). Fixed cells were imaged using a 63 × 1.4 NA oil objective (Leica) with the LAS X software (Leica) on the Leica TCS SP8 (Leica). Image stacks of 15 optical sections with a spacing of 0.21 μm through the cell volume were taken.

Following image acquisition, maximum-intensity projection of the fluorescent channels was performed in ImageJ v1.52j (NIH). Analysis of SGs in iN was performed by first using Ilastik (77) to identify and classify CHGA puncta signals, CHGB puncta signals, background signals, and neurite signals. Then, segmented images along with original images were analyzed in CellProfiler to quantify CHGA and CHGB colocalized puncta along neurite tracks.

### RNA preparation and qRT-PCR.

RNA was extracted from cultured cells using RNAStat60 (TelTest) according to the manufacturer’s instructions followed by DNase I (Roche) treatment. A total of 4 μg of total RNA was converted to cDNA using High Capacity cDNA Reverse Transcription kit (Life Technologies, Thermo Fisher Scientific) and a C1000 Thermal Cycler (Bio-Rad). Quantitative PCR reactions were run in triplicate in a QuantStudio 7 Real-Time PCR system (Applied Biosystems, Thermo Fisher Scientific). Analysis of results was done using the ΔΔCt method and normalized to GAPDH. Primers are listed in Supplemental Table 5.

### Human subjects.

Neurotypical control teeth were obtained through the Department of Pediatric Dentistry at the University of Tennessee Health Science Center (UTHSC). Teeth from children with neurogenetic disorders (PWS, AS, and SYS) were collected remotely by parents after normal exfoliation. The UTHSC Institutional Review Board approved this study. Subjects provided informed consent for tooth collection and storage of DPSCs in a deidentified repository. The Social Communication Questionnaire, which has been validated against the Autism Diagnostic Interview-Revised as a proxy for measuring potential ASD (78), was used to assess ASD status, as shown in Supplemental Table 3. All protocols and consenting procedures corresponding to Figure 8H were approved by the IRB of Columbia University Medical Center, as shown in Supplemental Table 4.

### Generation of DPSC cultures.

Immediately following loss of the tooth, it was placed in media (DMEM/F12 50/50 mix with HEPES) (Thermo Fisher Scientific), 100 U/mL penicillin (Thermo Fisher Scientific), and 100 μg/mL streptomycin (Thermo Fisher Scientific). DPSCs were isolated and cultured as previously described (41). Briefly, after mincing the dental pulp from inside the tooth cavity, 3 mg/mL Dispase II (Thermo Fisher Scientific) and 4 mg/mL Collagenase I (Thermo Fisher Scientific) were added to digest the tissue. Cells were then seeded on poly-d-lysine–coated (Thermo Fisher Scientific) 12-well plates with DMEM/F12 1:1, 10% (*v/v*) fetal bovine serum (Thermo Fisher Scientific), 10% (*v/v*) newborn calf serum (Thermo Fisher Scientific), 100 U/mL penicillin, and 100 μg/mL streptomycin. Then, 80% confluent cultures were passaged with Tryple E express (Gibco, Thermo Fisher Scientific) and neuronal differentiation performed only on early-passage cells (<4).

### Generation of DPSC-derived neurons and adipocytes.

DPSCs were seeded at 20,000 cells/cm^2^ on poly-d-lysine–coated plates or chamber slides (Ibidi) with DMEM/F12 1:1, 10% (*v/v*) fetal bovine serum (FBS), 10% (*v/v*) newborn calf serum, 100 U/mL penicillin, and 100 μg/mL streptomycin. At 80% confluence, the neuronal differentiation protocol was followed as previously described (41). Briefly, epigenetic reprogramming was performed by exposing the DPSCs to 10 μM 5-azacytidine (Acros Scientific) in DMEM/F12-containing 2.5% (*v/v*) FBS and 10 ng/mL basic FGF (bFGF) (Thermo Fisher Scientific) for 48 hours. Neural differentiation was induced by exposing the cells to 250 μM IBMX (Thermo Fisher Scientific), 50 μM forskolin (Thermo Fisher Scientific), 200 nM TPA (Thermo Fisher Scientific), 1 mM dibutyryl cAMP, 10 ng/mL bFGF (Thermo Fisher Scientific), 10 ng/mL nerve growth factor (Invitrogen, Thermo Fisher Scientific), 30 ng/mL NT-3 (PeproTech), and 1% (*v/v*) insulin-transferrin-sodium selenite premix (Thermo Fisher Scientific) in DMEM/F12 for 3 days. Neuronal maturation was performed by maintaining the cells in Neurobasal A media (Gibco, Thermo Fisher Scientific) with 1 mM dibutyryl cAMP, 2% (*v/v*) B27, 1% (*v/v*) N2 supplement, 30 ng/mL NT-3 (Gibco, Thermo Fisher Scientific), and 1× Glutamax (Gibco, Thermo Fisher Scientific) for 21 days.

DPSCs were differentiated into adipose cells as previously described (79). Briefly, DPSC cell lines were seeded at 20,000 cells/cm^2^ on poly-d-lysine–coated chamber slides. Once cells reached (80%) confluence, they were grown in adipogenic media (Lonza) for 21 days.

### Electron microscopy analysis.

Whole mice were perfused with a 2.5% (*v/v*) glutaraldehyde and 1.5% (*v/v*) paraformaldehyde fixative in 0.1 M phosphate buffer. Whole hypothalamus was removed and samples were contrasted successively with 1% (*v/v*) osmium tetroxide (Electron Microscopy Sciences) and 1% (*v/v*) uranyl acetate (Electron Microscopy Sciences) in distilled water and washed with distilled water between contrasting steps. Samples were then dehydrated by increasing alcohol concentration, infiltrated with EmBed 812 (Electron Microscopy Sciences), and polymerized at 80°C overnight. Samples were sectioned on a Leica ultramicrotome (Wetzlar) at 70 nm to isolate the medial eminence and examined in a Tecnai G2 F20-TWIN transmission electron microscope. Images were acquired using an AMT side mount camera system.

### ELISA analysis.

Mouse whole blood was collected by cardiac puncture with a 25-gauge needle and left undisturbed at room temperature for 1 hour to clot. Serum was collected by centrifugation 2000*g* for 15 minutes at 4°C. The following kits were used for ELISA experiments: OXT (RayBiotech, EIAM-OXT-1), AVP (RayBiotech, EIA-NEU2-1), SST (RayBiotech, EIAM-SOM-1), GnRH (MyBioSource, MBS160777), and MSH (MyBioSource, MBS263324). For iN ELISAs, standard iN growth media were collected alone or 60 minutes following 50 mM KCl (Thermo Fisher Scientific) treatment at 37°C. Media was centrifuged at 16,000*g* for 15 minutes at 4°C. Protein concentration was measured by BCA assay before analysis by oxytocin ELISA (MyBioSource, MBS160452).

For ELISAs on human material, venous blood samples were collected from individuals with genetically confirmed PWS and unaffected nuclear family controls. All subjects fasted overnight, with the last meal 10–12 hours before the blood draw. Blood was obtained from each nuclear family member within approximately a 1-hour time window. Whole blood was collected into K2 EDTA tubes (BD 367862) and was spun down at 2000*g* for 20 minutes at 4°C. CHGB concentration was assayed using the LSBio Human CHGB/Chromogranin B Sandwich ELISA Kit. Samples were run in triplicate in a blinded fashion by Quansys Biosciences (Logan, Utah, USA). As recommended by the manufacturer, plasma samples were diluted 1:10 in sample buffer.

### Statistics.

All bar graphs display the mean ± SD, with experimental groups normalized to control group and each data point representing a unique animal, individual, or induction experiment. Statistical analysis was performed by unpaired 2-tailed *t* test or 1-way ANOVA followed by Bonferroni’s test, with *P* value indicated as *P < 0.05, **P < 0.01, ***P < 0.001, and ****P < 0.0001.

### Study approval.

All mouse and human samples used in this study were obtained with the approval of IRBs of participating institutions (Columbia University Medical Center and UTHSC). All animal work was carried out with approval of the IACUC of participating institutions (St. Jude Children’s Research Hospital and Mayo Clinic). Informed consent was obtained from the parents or legal guardians of all patients.

## Author contributions

HC and PRP conceived and designed the study. HC and JK acquired data, performed experiments, and analyzed all data (unless listed below). AKV collected and generated human DPSC lines. HC, JKD, and KFT collected *Magel2* mouse samples. KFT performed electron microscopy and analyzed electron microscopy data. HST performed IHC analysis. DJCT, CDE, and AN generated iPSC lines. LCB, MR, and YZ performed human CHGB ELISA and analyzed human CHGB ELISA data. LD collected *Washc1* mouse samples. HC and JT performed immunofluorescence image analysis. JJM and JKD performed proteomics and analyzed proteomics data. HC, JRY, RLL, MET, DDB, LTR, and PRP acquired funding for and supervised this study. HC and PRP wrote the manuscript with input from all authors.

## Supplementary Material

Supplemental data

Supplemental Table 1

Supplemental Table 2

## Version 1 09/03/2020

Electronic publication
