# Supplementary material for: Loss of MAGEL2 in Prader-Willi syndrome leads to decreased secretory granule and neuropeptide production
Source: JCI Insight. 2020 Sep 3;5(17):e138576. doi: 10.1172/jci.insight.138576 (PMC7526459; doi:10.1172/jci.insight.138576)

Supplemental Material

Supplemental Methods

Lentiviral vector production and titration

SJ293TS cells was transfected with pCAG-kGP1-1R, pCAG-VSVG and pCAG4-RTR2 using PEIpro (Polyplus Transfection). The next day, transfected cells were diluted with media containing 12.5 U/mL Benzonase (Sigma). Vector supernatants were collected 48 hr post-transfection, clarified by centrifugation at 330 xg for 5 min and filtered. Lentiviral vector containing supernatants were adjusted to 300 mM NaCl, 50 mM Tris pH 8.0 and loaded onto an Acrodisc Mustang Q membrane (Pall Life Sciences) according to the manufacturer's instructions using an Akta Avant chromatography system (GE Healthcare). After washing the column 300 mM NaCl, 50 mM Tris pH 8.0, viral particles were eluted from the column using 2 M NaCl, Tris pH 8.0 directly onto a PD10 desalting column (GE Healthcare) according to the manufacturer's instructions. Vector containing flow-through was diluted with an equal volume of X-VIVO 10 media (Lonza) or phosphate buffered saline containing 0.5% (v/v) human serum albumin (Grifols Biologics) to achieve an approximate 50-fold concentration from the starting material, sterile filtered, aliquoted and stored at −80 °C.

Titration of lentiviral vectors was performed by transducing HOS cells with serially diluted vector preparations in the presence 5-8 mg/mL Polybrene (Sigma). Four days post-transduction, genomic DNA was isolated from transduced HOS cells using a Quick-DNA Miniprep kit (Zymo Research). Vector titers were determined by calculating the ratio between the copies of HIV psi and every two copies of RPP30 via QX200 digital droplet PCR system (Bio-Rad), multiplied by the number of cells transduced and if necessary, multiplied by the dilution factor.

Quantitative Proteomic / Mass Spectrometry

For adrenal gland, pituitary, liver, brain stem and WAT: the TMT labeled samples were analyzed on an Orbitrap Fusion Lumos mass spectrometer (ThermoFisher). Samples were injected directly onto a 25 cm, 100 µm ID column packed with BEH 1.7 µm C18 resin (Waters). Samples were separated at a flow rate of 400 nL/min on EASY-nLC (ThermoFisher). Buffer A and B were 0.1% (v/v) formic acid in water and acetonitrile, respectively. A gradient of 1–25% B over 180 min, an increase to 40% B over 40 min, an increase to 90% over 10 min and held at 90% B for 10 min was used for a 240 min total run time. The column was re-equilibrated with 20 μL of buffer A prior to the injection of the sample. Peptides were eluted directly from the tip of the column and nanospray directly into the mass spectrometer by application of 2.8 kV voltage at the back of the column. The Lumos was operated in a data-dependent mode. Full MS1 scans were collected in the Orbitrap at 120k resolution. The cycle time was set to 3 s, and within this 3 s the most abundant ions per scan were selected for CID MS/MS in the ion trap. MS3 analysis with multi-notch isolation (SPS3) was utilized for detection of TMT reporter ions at 60k resolution. Monoisotopic precursor selection was enabled and dynamic exclusion was used with an exclusion duration of 10 sec.

For hypothalamus: the TMT labeled samples were analyzed on an Orbitrap Fusion Tribrid mass spectrometer (Thermo Fisher). Samples were injected directly onto a 25 cm, 100 µm ID column packed with BEH 1.7 µm C18 resin (Waters). Samples were separated at a flow rate of 300 nL/min on EASY-nLC (ThermoFisher). Buffer A and B were 0.1% formic acid in water and acetonitrile, respectively. A gradient of 1–25% B over 200 min, an increase to 50% B over 120 min, an increase to 90% over 30 min and held at 90% B for 10 min was used for a 360 min total run time. The column was re-equilibrated with 20 μL of buffer A prior to the injection of the sample. Peptides were eluted directly from the tip of the column and nanospray directly into the

mass spectrometer by application of 2.8 kV voltage at the back of the column. The Fusion was operated in a data-dependent mode. Full MS1 scans were collected in the Orbitrap at 120k resolution. The cycle time was set to 3 s, and within this 3 s the most abundant ions per scan were selected for CID MS/MS in the ion trap. MS3 analysis with multi-notch isolation (SPS3) was utilized for detection of TMT reporter ions at 60k resolution. Monoisotopic precursor selection was enabled and dynamic exclusion was used with an exclusion duration of 10 sec.

For peptide identification, tandem mass spectra were searched against a database including the UniProt mouse database one entry per gene (21982 entries released 12/1/2019) with common contaminants and reversed sequences using ProLuCID (69). The search was set with 50 and 600 ppm for precursor and fragments mass tolerance, respectively. The precursor mass range was set from 600 to 6000 with half or fully tryptic status. The N-term static modification was considered as (+229.1629) for TMT labeling and the amino acid residue specific static modifications were (+57.02146) on cysteine for carbamidomethylation and (+229.1629) on lysine for TMT labeling. Data was filtered using DTASelect with the following parameters, -p 1 -y 2 --trypstat --pfp 0.01 --extra --pI -DM 10 --DB --dm -in -t 1 --brief –quiet.

Supplemental Figures

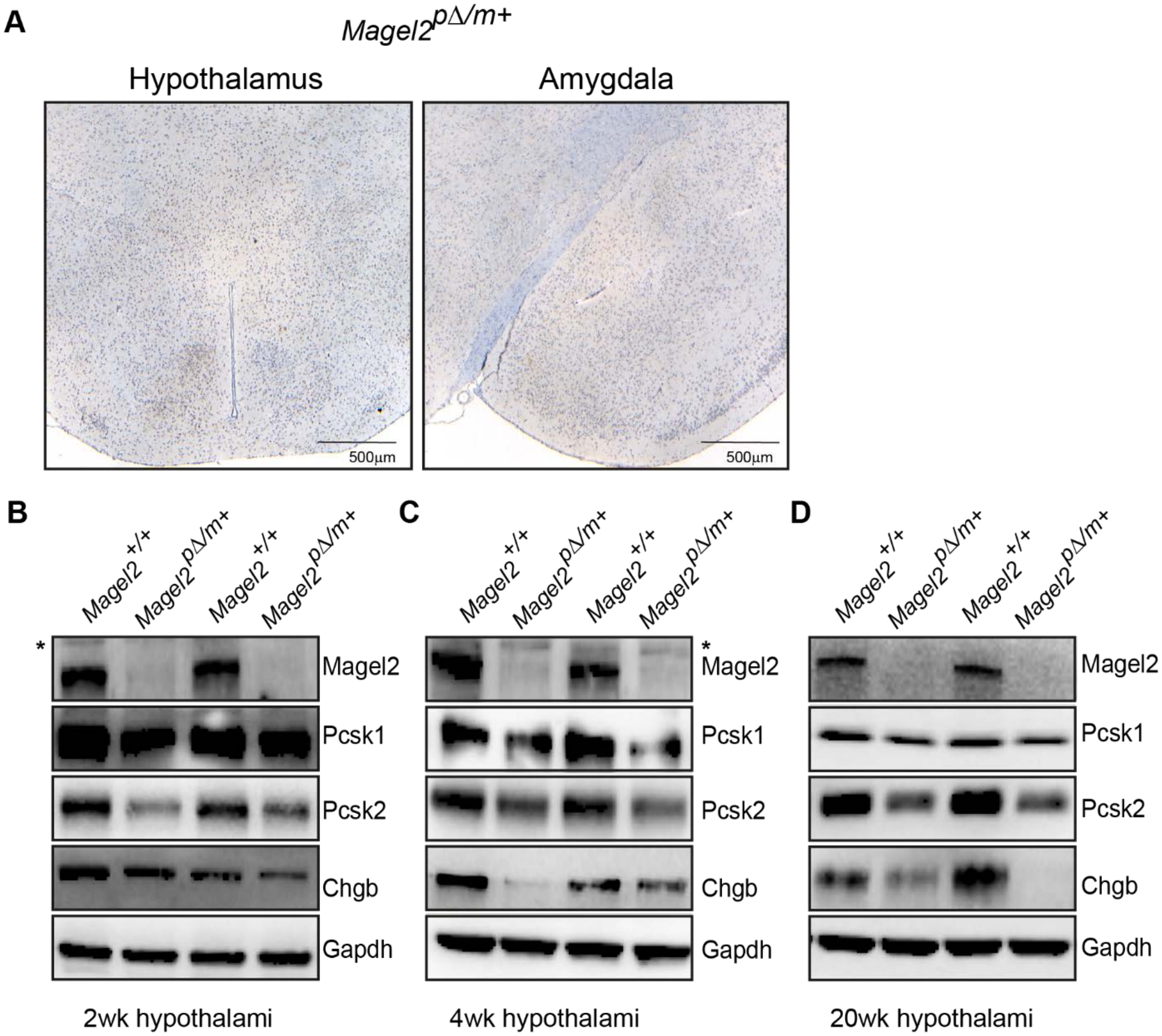

Figure S1. Western blot analysis of *Magel2*$^{+/+}$ and *Magel2*$^{p\Delta/m+}$ mouse hypothalami at 2, 4 and 20wks. A) Immunohistochemistry of 8wk old *Magel2*$^{p\Delta/m+}$ mouse at the hypothalamus and amygdala using anti-Magel2 antibody. **B-D)** Western blot analysis confirmed reduced expression of Pcsk1, Pcsk2 and Chgb in **B)** 2wk; **C)** 4wk; and **D)** 20wk old *Magel2*$^{p\Delta/m+}$ mouse hypothalami. Asterisks mark a non-specific band detected by Magel2 antibody. Gapdh served as loading control.

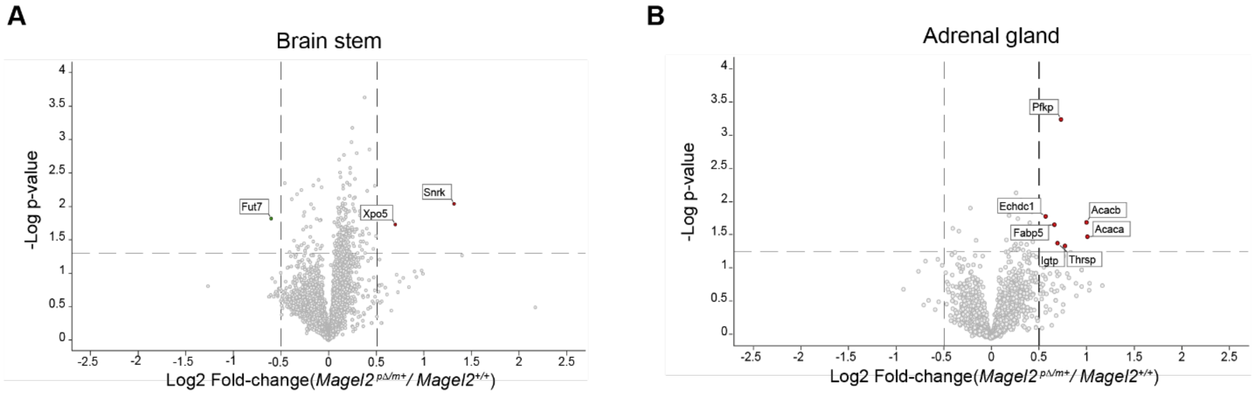

Figure S2. Quantitative proteomics of brain stem and adrenal gland in *Magel2*$^{p\Delta/m+}$ mice. Volcano plots showing proteins that are significantly changed in 8wk old *Magel2*$^{p\Delta/m+}$ mouse **A)** brain stem; and **B)** adrenal glands detected by TMT-M/S. The horizontal lines denoting p-value thresholds ($p \leq 0.05$; unpaired, analyzed by two-tailed student t-test) and vertical lines denoting log2 fold-change thresholds (>0.5 and <-0.5) n=5 per genotype.

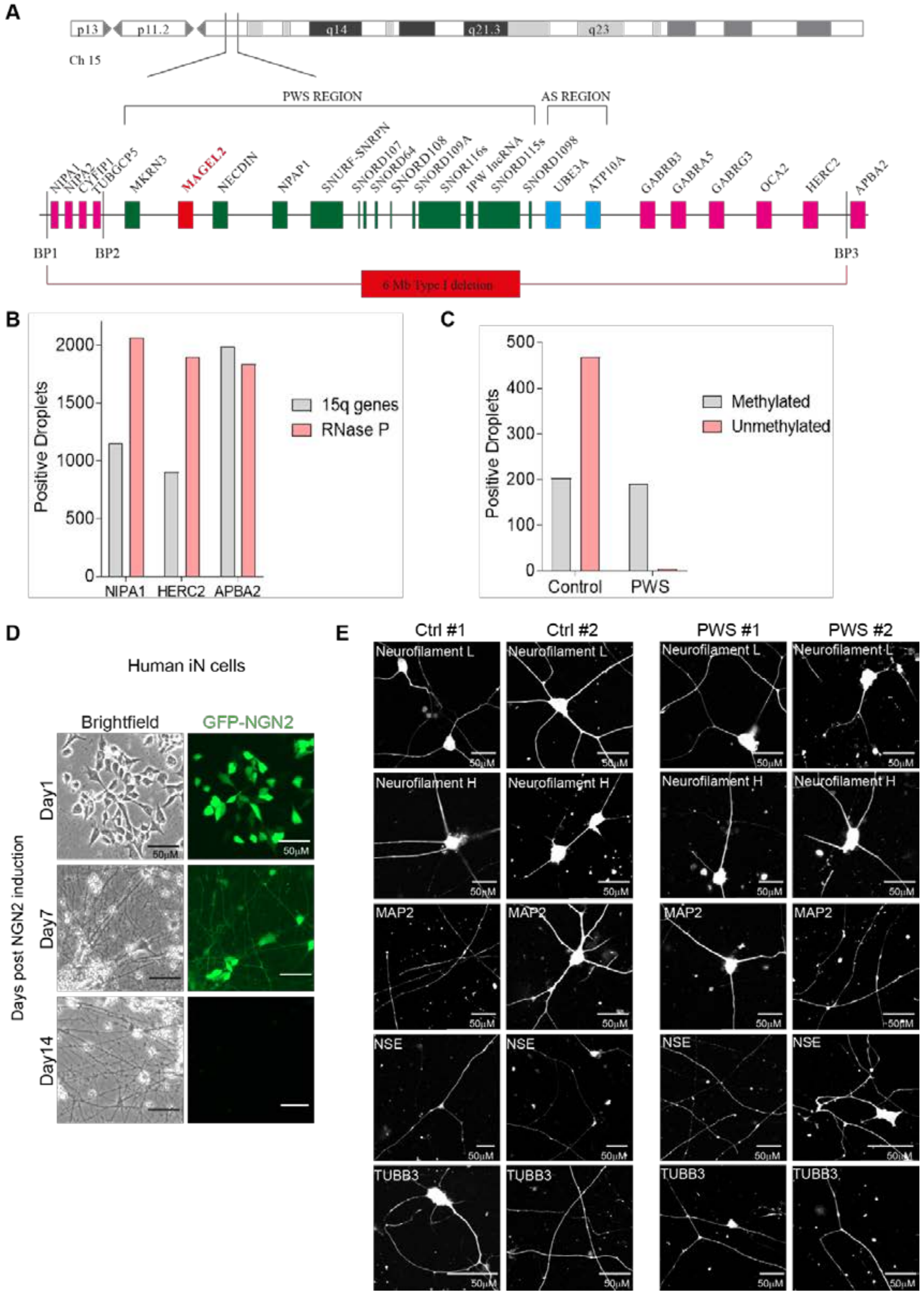

Figure S3. Characterization of *NGN2*-generated human iN. A) Schematic of chromosome 15 PWS region and genomic editing. **B)** Representative images showing the time course of conversion of human iPSC into iN by GFP-*NGN2*. **C)** Representative images of immunofluorescence staining of control and PWS iN with various neuronal markers. Scale bars=50 µm.

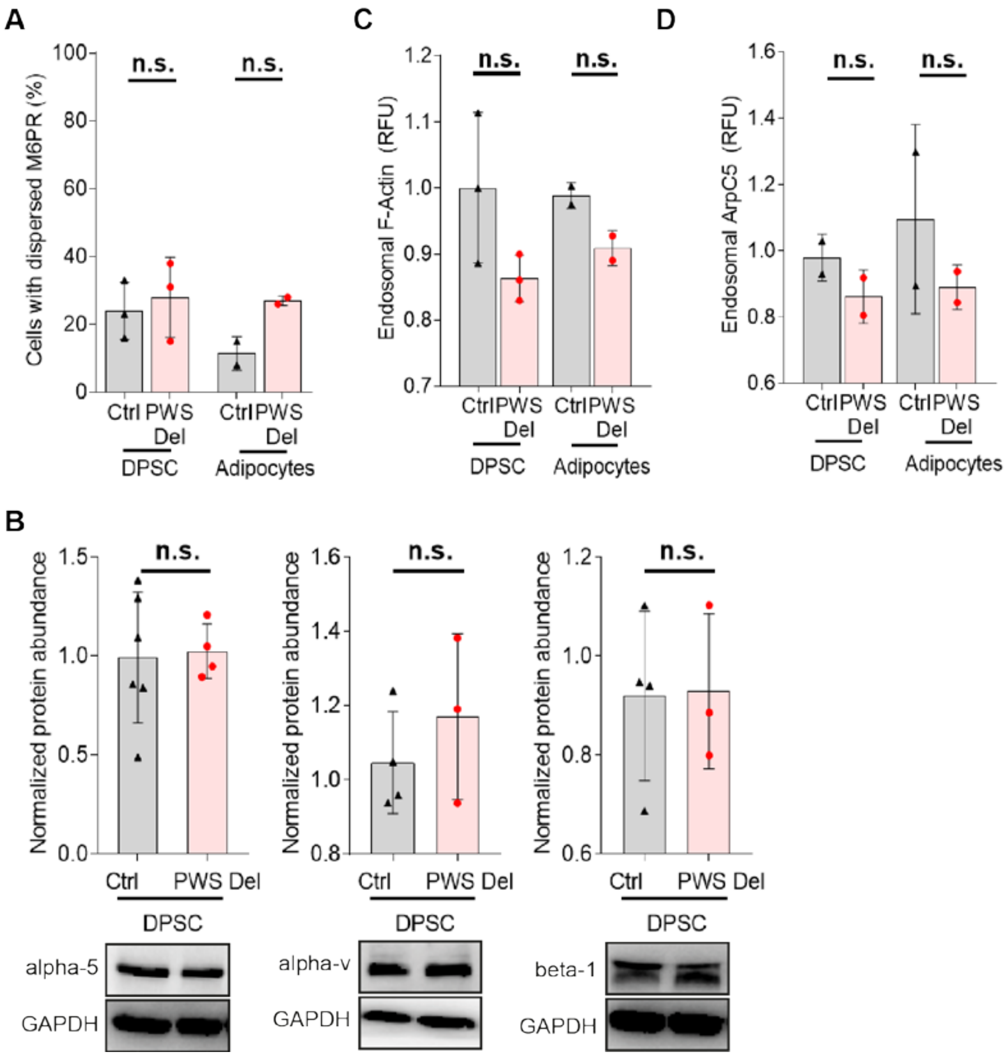

Figure S4. Endosomal protein recycling is not impaired in undifferentiated DPSC and DPSC-derived adipocytes. A) There is no difference in proportion of cells with impaired M6PR trafficking between control and PWS deletion DPSC and DPSC-derived adipocytes. Each data point represents one individual, plotted as mean±SD; >75 cells/data point, and analyzed by t-test. **B)** Quantification of western deletion analysis showed no difference in the expression of alpha-5, alpha-V and beta-1 between control and PWS deletion DPSC. Each target protein is first normalized to GAPDH, and then PWS deletion DPSC is normalized to averaged control. Each data point represents one individual, plotted as mean±S.D. and analyzed by unpaired, two-tailed student t-test. **C-D)** There is no difference in the fluorescence intensity of F-actin **(C)** or ArpC5 **(D)** on VPS35-marked endosomes between control and PWS deletion DPSC, and DPSC-derived adipocytes. Each data point represents one individual, plotted as mean±SD; >75 cells/data point, and analyzed by unpaired, two-tailed student t-test.

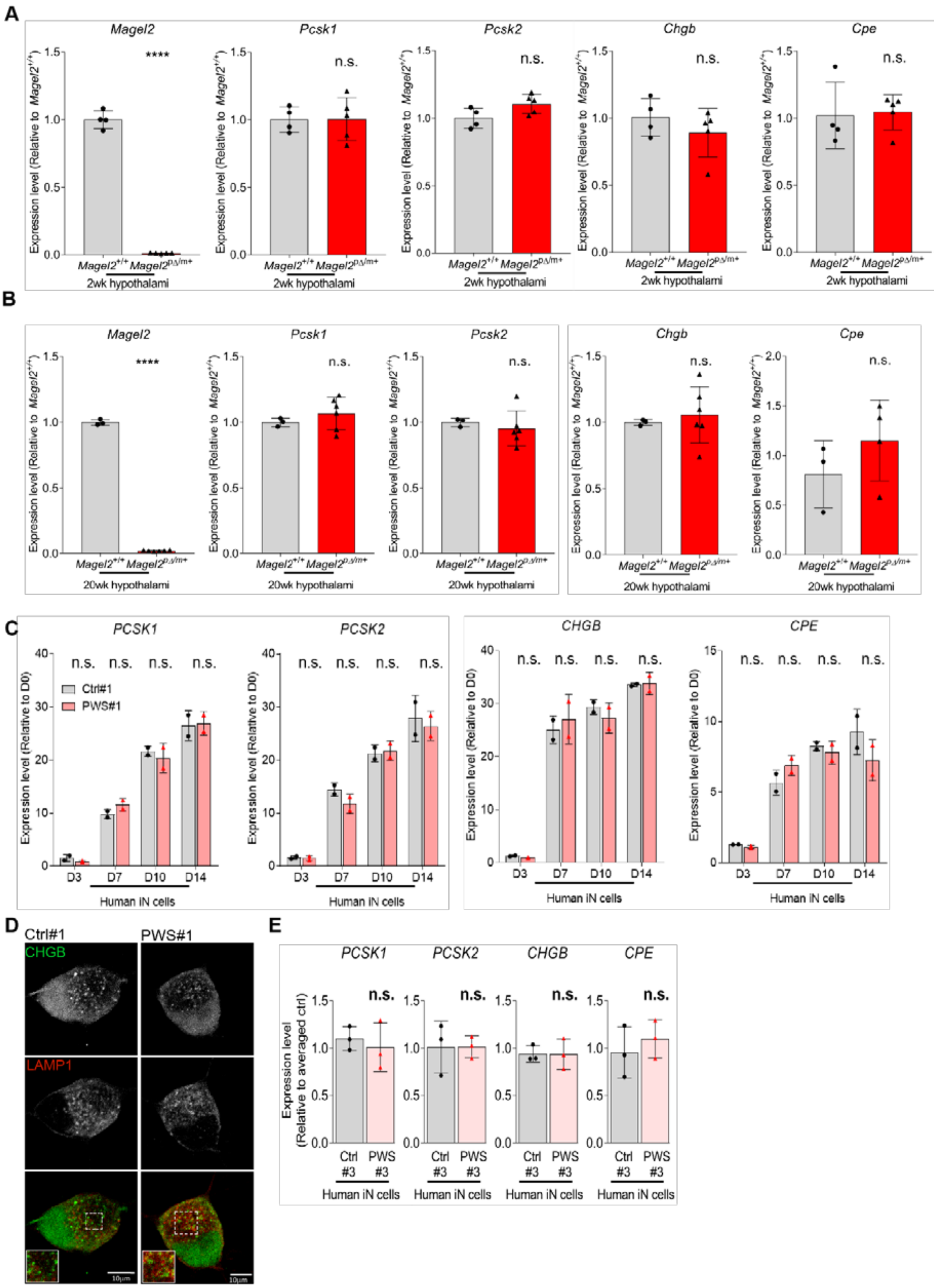

Figure S5. SG protein transcript levels are not altered in *Magel2*$^{p\Delta/m+}$ mice and PWS human iN. A-B) Transcript levels of *Pcsk1*, *Pcsk2*, *Chgb* and *Cpe* are unaltered between 2wk **(A)** and

20wk **(B)** old *Magel2*$^{p\Delta/m+}$ and *Magel2*$^{+/+}$ mouse hypothalami. Each data point represents one animal, plotted as mean±S.D. and analyzed by unpaired, two-tailed student t-test, ****p<0.0001. (n>3 per genotype) **C)** Transcript levels of *PCSK1*, *PCSK2*, *CHGB* and *CPE* are unaltered between control and PWS iN at 3d, 7d, 10d and 14d post-induction. Each data point represents one induction experiment (n=2), plotted as mean±S.D. and analyzed by ANOVA. **D)** Representative images of immunofluorescence staining with CHGB and LAMP1 in control and PWS iN. **E)** Transcript levels of *PCSK1*, *PCSK2*, *CHGB* and *CPE* are unaltered between control #3 and PWS #3 iN at 14d post-induction. Each data point represents one induction experiment (n=3), plotted as mean±S.D. and analyzed by t-test.

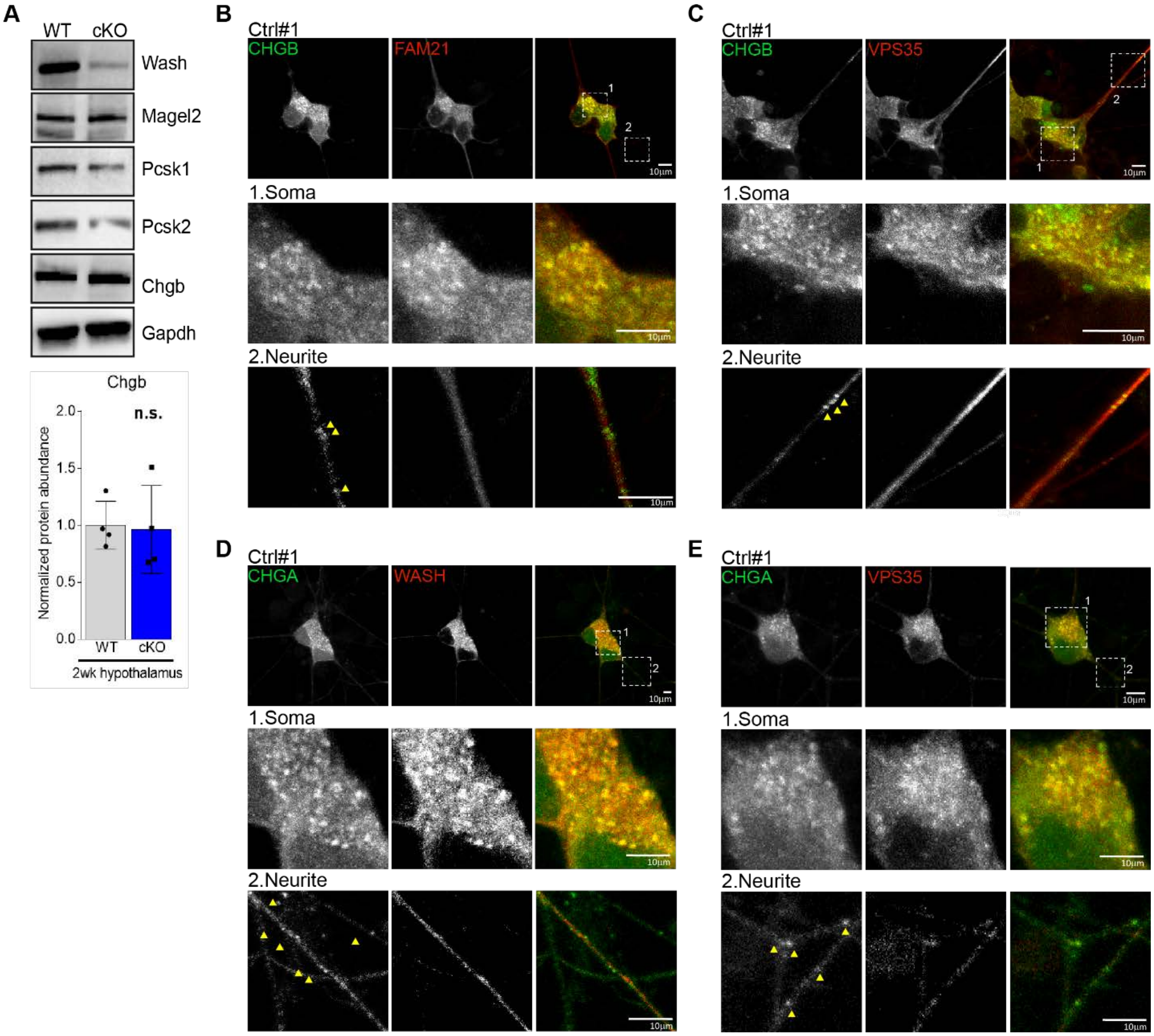

Figure S6. SG proteins colocalize with WASH and retromer components within the soma of human iN. A) Western blot analysis of Pcsk1, Pcsk2, Chgb and Magel2 in 2wk old *Washc1* cKO mouse hypothalami. Gapdh served as loading control. Each data point represents one animal, n=4 per genotype, plotted as mean±S.D. and analyzed by unpaired, two-tailed student t-test. **B)** Representative images of immunofluorescence staining with CHGB and FAM21 at both soma and neurite in control iN. Scale bars=10 µm. **C)** Representative images of immunofluorescence staining with CHGB and VPS35 at both soma and neurite in control iN. Scale bars= 10 µm. **D)** Representative images of immunofluorescence staining with CHGA and WASH at both soma and neurite in control iN. Scale bars represent 10 µm. **E)** Representative images of immunofluorescence staining with CHGA and VPS35 at both soma and neurite in control iN. Scale bars=10 µm.

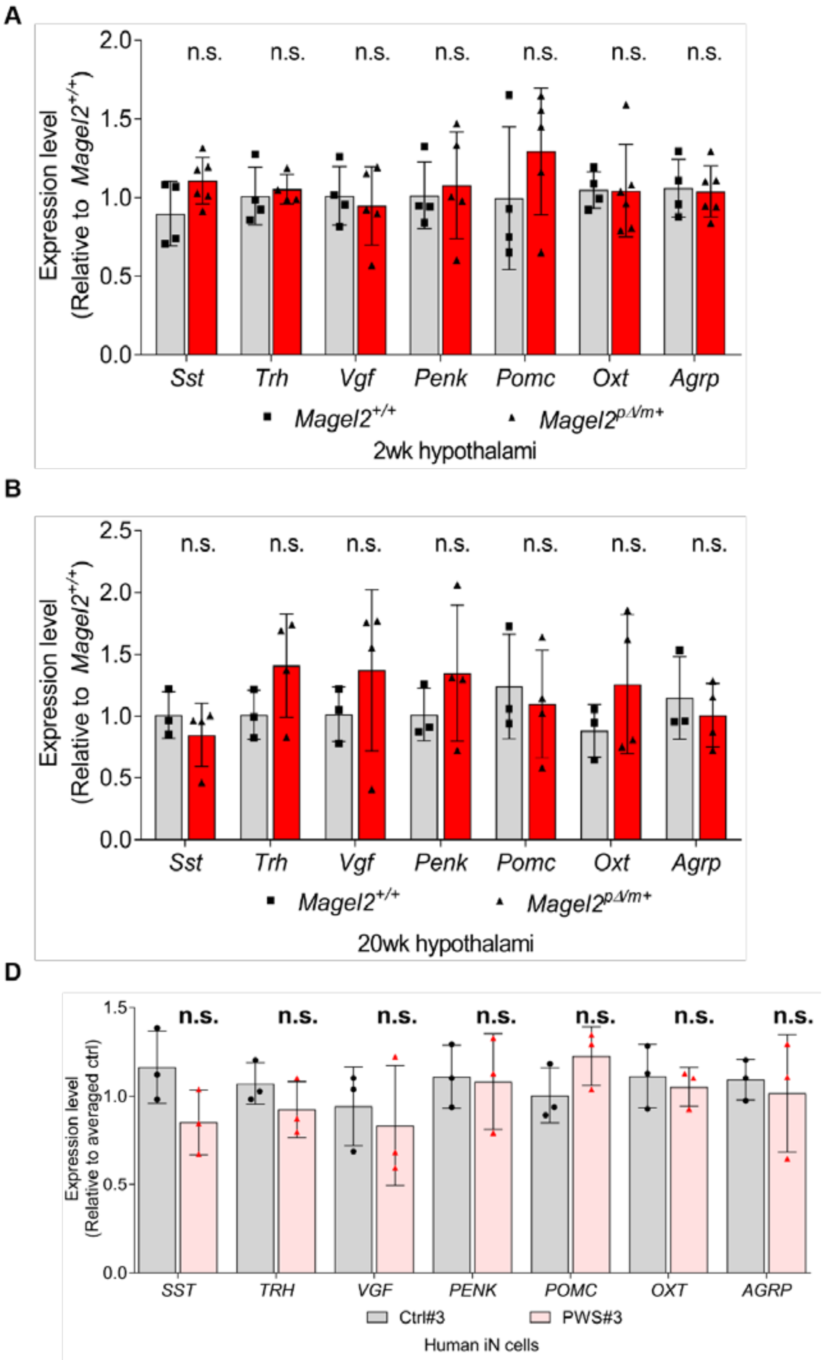

Figure S7. Neuropeptide transcript levels are not altered in *Magel2*$^{p\Delta/m+}$ mice. Transcript levels of various neuropeptides between 2wk **(A)** and 20wk **(B)** old *Magel2*$^{p\Delta/m+}$ and *Magel2*$^{+/+}$ mouse hypothalami. Each data point represents one animal, (n>3 per genotype), plotted as mean±S.D. and analyzed by unpaired, two-tailed student t-test. **C)** Transcript levels of various neuropeptides between Ctrl#3 and PWS#3 iN at 14d post-induction. Each data point represents one induction experiment (n=3), plotted as mean±S.D. and analyzed by t-test.

Supplemental Tables

Table S1. Protein expression differences between Magel2$^{+/+}$ (WT) and Magel2$^{p-/m+}$ (KO) (n=5 each) as determined by TMT proteomics for the indicated tissues.

Attached .xls file

Table S2. Gene ontology (GO) analysis of TMT proteomics data for the indicated tissues.

Attached .xls file

Table S3. Information on Prader-Willi and Schaaf-Yang syndrome patients and control subjects in which DPSCs were created from.

Cell Line	Genotype	Sex	Age (Years)	Autism Status
48	Control	M	7	Possible ASD
50	Control	M	unknown	unknown
195	Control	M	4.4	Normal
238	Control	M	8.3	Normal
302	Control	M	9.9	Normal
38	Control	F	10.3	unknown
76	Control	F	4.3	unknown
182	Control	F	12.8	Normal
188	Control	F	5.2	Normal
189	Control	F	8.4	unknown
233	Control	F	11.9	Normal
312	Control	F	8	Normal
148	PWS, deletion	M	5.8	Normal
152	PWS, deletion	M	5.4	Normal
198	PWS, deletion	M	8	Normal
225	PWS, deletion	M	11.6	Normal
297	PWS, deletion	M	5.6	Normal
192	PWS, deletion	F	13.3	Normal
258	PWS, deletion	F	5.5	Normal
235	PWS, UPD	M	10.1	Normal
94	PWS, UPD	F	5.1	Possible ASD
162	PWS, UPD	F	4.6	Normal
249	PWS, UPD	F	8.1	Normal
268	PWS, UPD	F	6.6	Possible ASD
270	PWS, UPD	F	4.6	Normal
277	PWS, UPD	F	16.2	Possible ASD
306	Schaaf-Yang	F	7	Possible ASD
274	Schaaf-Yang	F	7.2	Possible ASD

129	Angelman, deletion	M	12.8	Possible ASD
130	Angelman, deletion	M	6.5	unknown
78	Angelman, deletion	F	6.8	unknown

Table S4. Information on PWS patients and control subjects included in Fig. 8J.

**Sample ID**	**Genotype**	**Familial status**	**Gender**	**Age (YRS)**
0030 - CU	Unaffected Control	Parent	Female	34.0
0031 - CU	Unaffected Control	Parent	Male	34.3
0034 - CU	Unaffected Control	Parent	Female	30.6
0035 -CU	Unaffected Control	Parent	Male	32.7
0037 - CU	Unaffected Control	Sibling	Female	1.7
0038 - CU	Unaffected Control	Parent	Female	38.0
0039 - CU	Unaffected Control	Parent	Male	40.3
0041 - CU	Unaffected Control	Sibling	Female	3.7
0042 - CU	Unaffected Control	Parent	Female	35.3
0043 - CU	Unaffected Control	Parent	Male	35.3
0045 - CU	Unaffected Control	Sibling	Female	9.2
0046 - CU	Unaffected Control	Parent	Female	39.3
0047 - CU	Unaffected Control	Parent	Male	43.4
0049 - CU	Unaffected Control	Parent	Female	30.3
0050 - CU	Unaffected Control	Parent	Male	33.4
0052 - CU	Unaffected Control	Sibling	Male	4.5
0053 - CU	Unaffected Control	Sibling	Female	1.6
0054 - CU	Unaffected Control	Parent	Female	39.4
0055 - CU	Unaffected Control	Parent	Male	40.8
0056 - CU	Unaffected Control	Sibling	Male	1.5
0058 - CU	Unaffected Control	Parent	Male	48.7
0060 - CU	Unaffected Control	Parent	Female	47.0
0061 - CU	Unaffected Control	Parent	Male	48.2
0063 - CU	Unaffected Control	Sibling	Female	11.5
0064 - CU	Unaffected Control	Sibling	Female	6.4
0065 - CU	Unaffected Control	Parent	Male	38.2
0067 - CU	Unaffected Control	Parent	Female	44.2
0068 - CU	Unaffected Control	Parent	Male	54.7
0070 - CU	Unaffected Control	Sibling	Male	5.1
0032 - CU	PWS Deletion	Proband	Female	5.6
0036 - CU	PWS Deletion	Proband	Male	3.1
0040 - CU	PWS Deletion	Proband	Female	1.6
0044 - CU	PWS UPD	Proband	Female	6.5
0048 - CU	PWS Deletion	Proband	Male	5.2
0051 - CU	PWS Deletion	Proband	Male	6.1

0057 - CU	PWS UPD	Proband	Female	3.2
0059 - CU	PWS	Proband	Female	7.7
0062 - CU	PWS UPD	Proband	Male	8.4
0066 - CU	PWS UPD	Proband	Male	6.5
0069 - CU	PWS UPD	Proband	Male	2.3

Table S5. Human (Hu) and mouse (Mo) qRT-PCR primers used in this study.

Primer Name	Sequence
Hu MAP2 F	CCACCTGAGATTAAGGATCA
Hu MAP2 R	GGCTTACTTTGCTTCTCTGA
Hu DCX F	TATGCGCCGAAGCAAGTCTCCA
Hu DCX R	CATCCAAGGACAGAGGCAGGTA
Hu SST F	CCAGACTCCGTCAGTTTCTGCA
Hu SST R	TTCCAGGGCATCATTCTCCGTC
Hu TRH F	GGTGAGCACTCCGCGTC
Hu TRH R	TGTGCTGGGTTACATCGACT
Hu VGF F	ACAAGGTGGTTTTCGTCCGT
Hu VGF R	TGGCTTCTCGCTTCCATCAG
Hu PENK F	GCTTTTCCAATTGGCCTGCT
Hu PENK R	ATTCCATTACGCAAGCCAGGA
Hu POMC F	TACGGCGGTTTCATGACCTC
Hu POMC R	GCGTTCTTGATGATGGCGTT
Hu OXT F	GGTTGTTGAACAGAGCTCCACC
Hu OXT R	GGCAGTTCTGGATGTAGCAGG
Hu AGRP F	ACAACTGCAGAACAGGCAGAA
Hu AGRP R	TGCAGGTCTAGTACCTCTGCCAA
Hu PCSK1 F	TGATCCCACAAACGAGAACA
Hu PCSK1 R	ACCAGGTGCTGCATATCTCG
Hu PCSK2 F	AGAGAGGGCCATGCAATTC
Hu PCSK2 R	AGGTCTTCTCTCACCTCCTTCA
Hu CHGB F	CAGCCAACGCTGCTTCTCAG
Hu CHGB R	CAGCGAGTCACCATTCCTTCA
Hu CPE F	TAAATTCAGGCTCACCAGGC
Hu CPE R	CCATCAGCAGGATTTACACG
Hu MAGEL2 F	AAATGCGTTGGTGCAGTTCC
Hu MAGEL2 R	CCAGCTTATTGTTGGCACGG
Hu GAPDH F	GTCTCCTCTGACTTCAACAGCG
Hu GAPDH R	ACCACCCTGTTGCTGTAGCCAA
Mo Sst F	ACCCCAGACTCCGTCAGTT
Mo Sst R	TACTTGGCCAGTTCCTGTTTCC
Mo Trh F	GGTGCTGCCTTAGATTCCTG
Mo Trh R	CTTGTCTTGGTTGGCACGTC
Mo Vgf F	AGTCAGACCCATAGCCTCCC
Mo Vgf R	GCTTAGCATTGCTCGGACTG

Mo Penk F	GCGACATCAATTTCCTGGCGTG
Mo Penk R	CCTTGCAGGTCTCCCAGATTT
Mo Pomc F	GCTTGCAAACTCGACCTCTC
Mo Pomc R	TTCCGGGGGTTTTCAGTCAG
Mo Oxt F	GGAGAACTACCTGCCTTCG
Mo Oxt R	GTATTCCCAGAAAGTGGGCT
Mo Agrp F	CATCTTCCACCTTTGCAGCATT
Mo Agrp R	AGCATGGCCTTTGCTTGCTG
Mo Pcsk1 F	TGGAGTTGCATATAATTCCAAAGTT
Mo Pcsk1 R	AGCCTCAATGGCATCAGTTAC
Mo Pcsk2 F	GGCGTGTTTGCATTAGCTTT
Mo Pcsk2 R	GCACAGTCAGATGTTGCATGT
Mo Chgb F	CCTCTCAAATGCCCTATCCA
Mo Chgb R	CACCTTTGACCTCTTTTCCACT
Mo Cpe F	AAACTTACAGCCTCCGCTCC
Mo Cpe R	CAAGCTCAAAGTCCACCCCA
Mo Magel2 F	CTGGGAGATTCAGAGGGCTA
Mo Magel2 R	TGCGGAGTGTAGAGGGATTC
Mo Gapdh F	GTCTCCTCTGACTTCAACAGCG
Mo Gapdh R	ACCACCCTGTTGCTGTAGCCAA

Figure 1

A

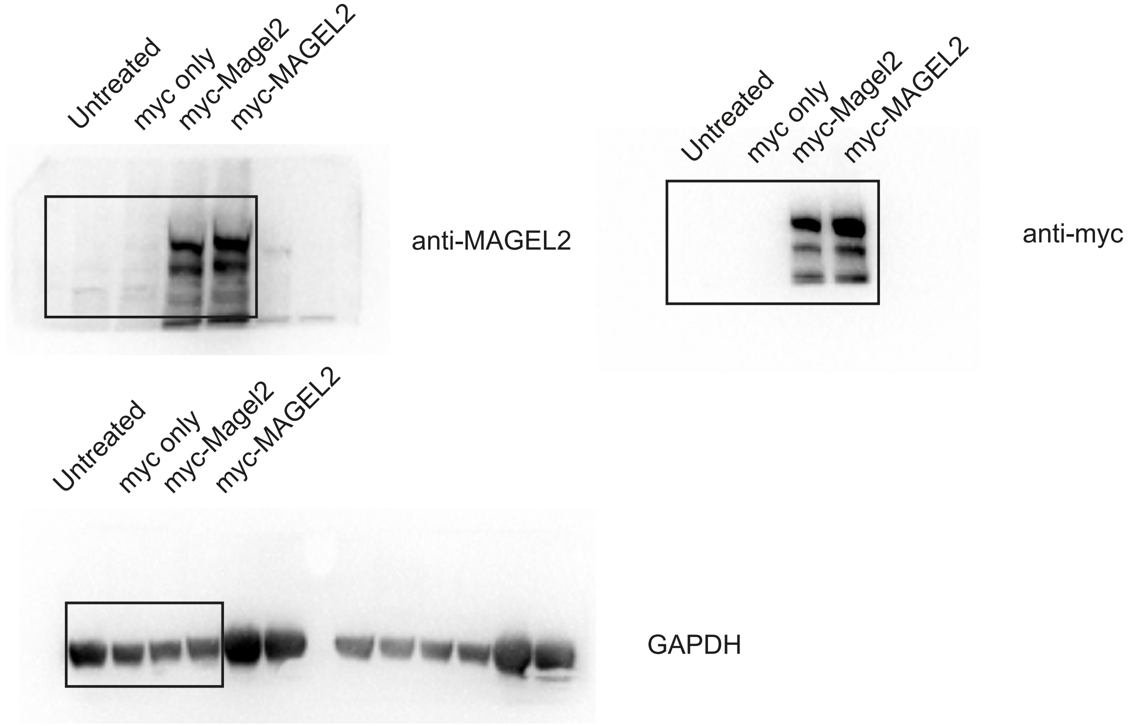
Untreated
myc only
myc-Magel2
myc-MAGEL2
anti-MAGEL2
Untreated
myc only
myc-Magel2
myc-MAGEL2
anti-myc
Untreated
myc only
myc-Magel2
myc-MAGEL2
GAPDH

B

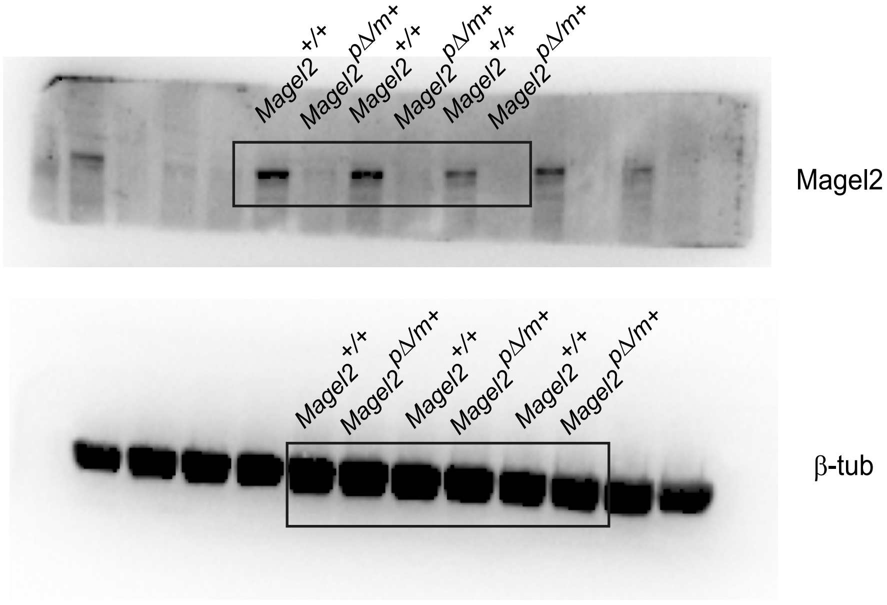
Magel2+/+
Magel2pΔ/m+
Magel2+/+
Magel2pΔ/m+
Magel2+/+
Magel2pΔ/m+
Magel2
Magel2+/+
Magel2pΔ/m+
Magel2+/+
Magel2pΔ/m+
Magel2+/+
Magel2pΔ/m+
β-tub

Figure 2

D

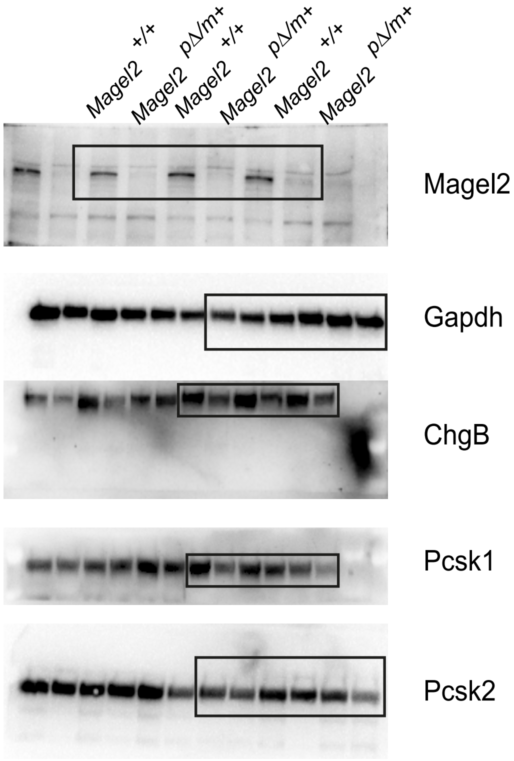
Magel2 +/+
Magel2 pΔ/m+
Magel2 +/+
Magel2 pΔ/m+
Magel2 +/+
Magel2 pΔ/m+
Magel2
Gapdh
ChgB
Pcsk1
Pcsk2

Figure 3

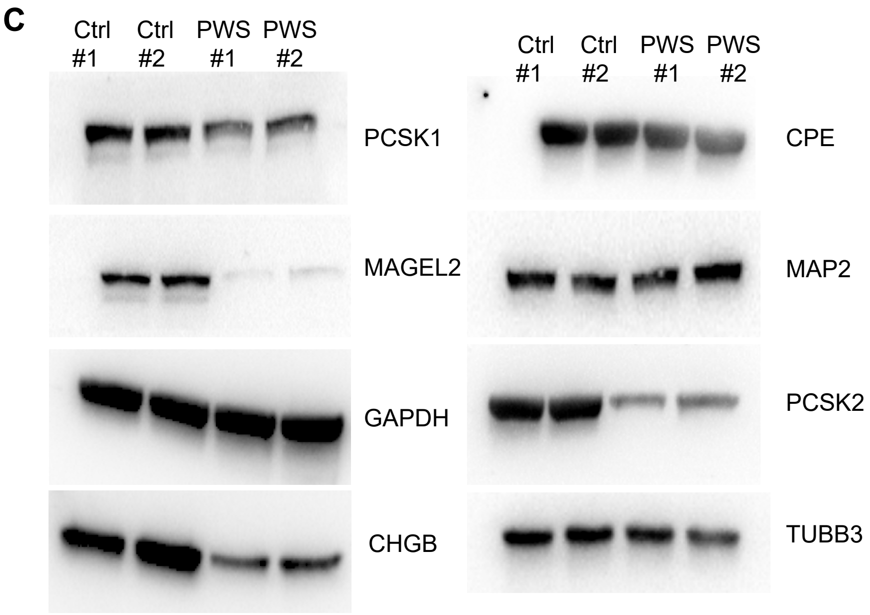
C
Ctrl #1
Ctrl #2
PWS #1
PWS #2
PCSK1
MAGEL2
GAPDH
CHGB
Ctrl #1
Ctrl #2
PWS #1
PWS #2
CPE
MAP2
PCSK2
TUBB3

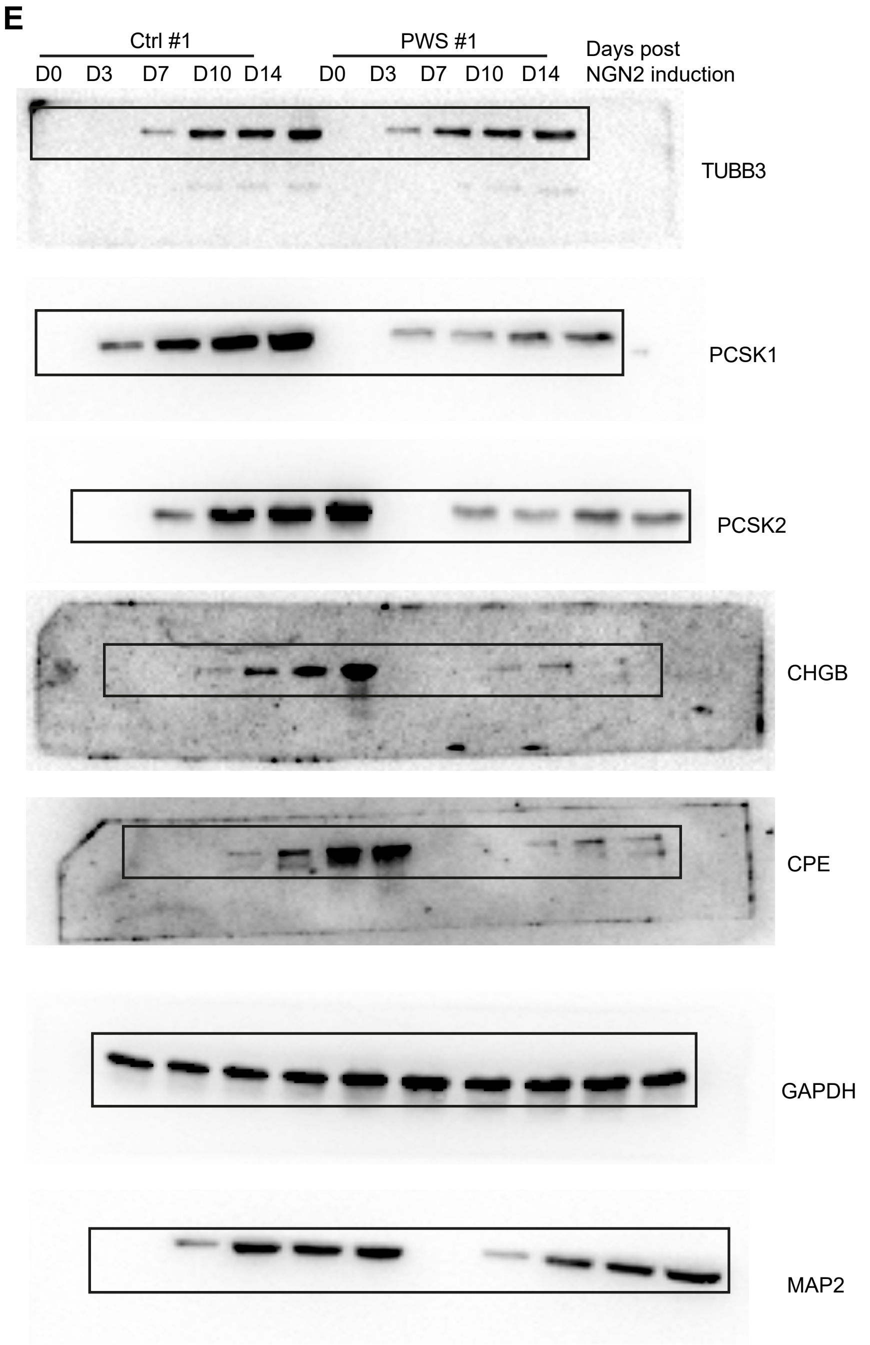
E
Ctrl #1
PWS #1
Days post NGN2 induction
D0 D3 D7 D10 D14 D0 D3 D7 D10 D14
TUBB3
PCSK1
PCSK2
CHGB
CPE
GAPDH
MAP2

Figure 4

D

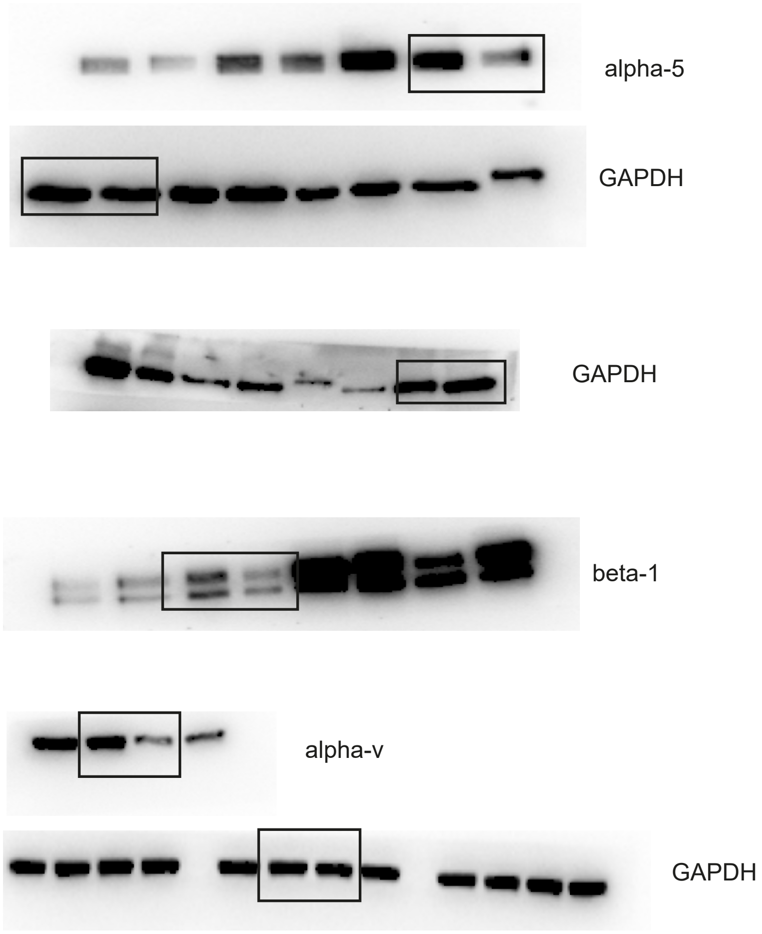
alpha-5
GAPDH
GAPDH
beta-1
alpha-v
GAPDH

H

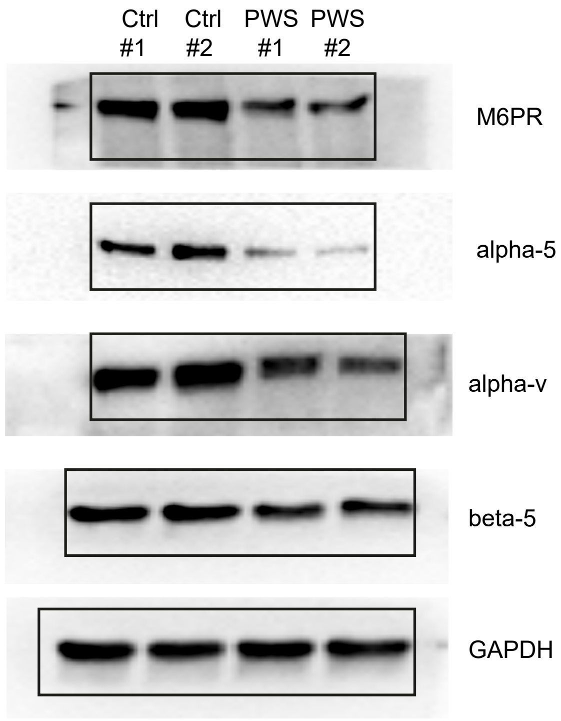
Ctrl #1
Ctrl #2
PWS #1
PWS #2
M6PR
alpha-5
alpha-v
beta-5
GAPDH

Figure 5

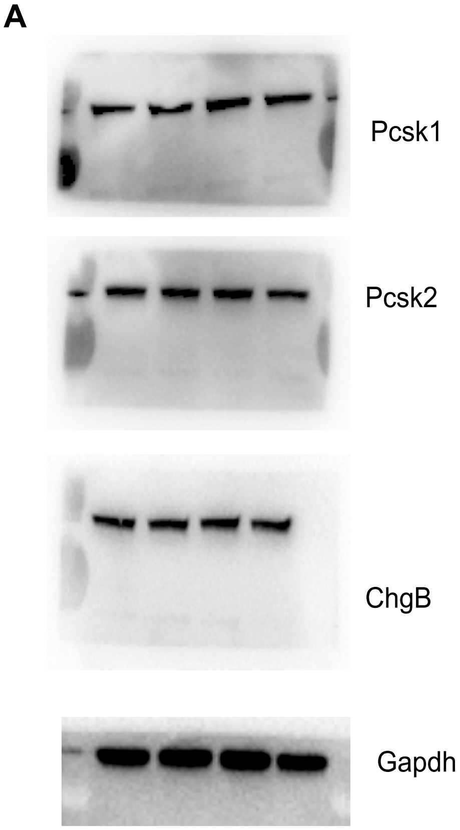

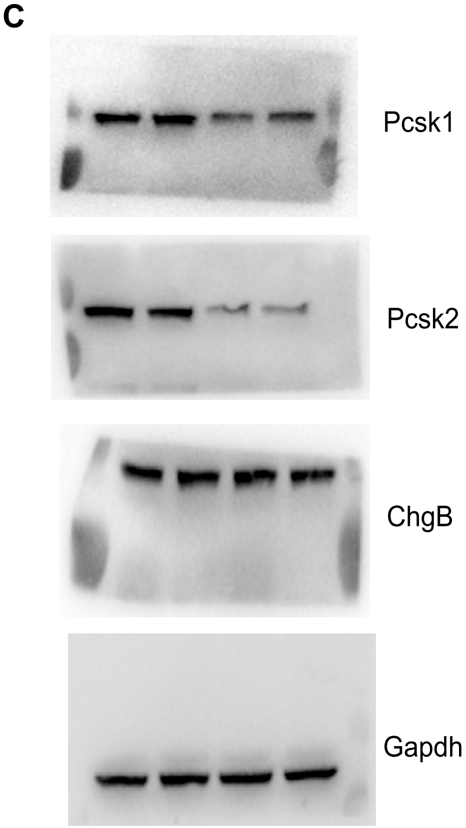

Figure 6

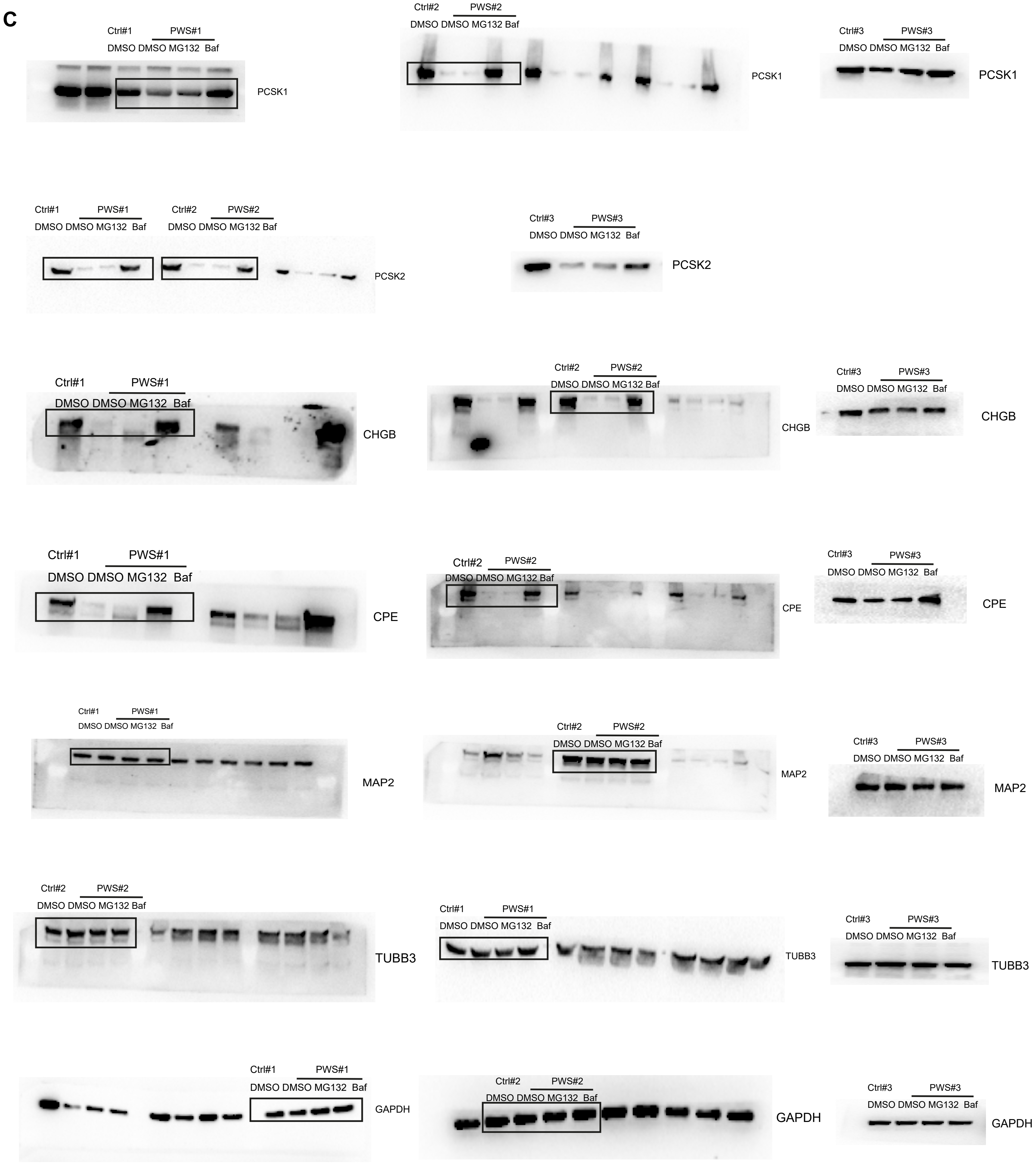
C
Ctrl#1 PWS#1
DMSO DMSO MG132 Baf
PCSK1
Ctrl#2 PWS#2
DMSO DMSO MG132 Baf
PCSK1
Ctrl#3 PWS#3
DMSO DMSO MG132 Baf
PCSK1
Ctrl#1 PWS#1 Ctrl#2 PWS#2
DMSO DMSO MG132 Baf DMSO DMSO MG132 Baf
PCSK2
Ctrl#3 PWS#3
DMSO DMSO MG132 Baf
PCSK2
Ctrl#1 PWS#1
DMSO DMSO MG132 Baf
CHGB
Ctrl#2 PWS#2
DMSO DMSO MG132 Baf
CHGB
Ctrl#3 PWS#3
DMSO DMSO MG132 Baf
CHGB
Ctrl#1 PWS#1
DMSO DMSO MG132 Baf
CPE
Ctrl#2 PWS#2
DMSO DMSO MG132 Baf
CPE
Ctrl#3 PWS#3
DMSO DMSO MG132 Baf
CPE
Ctrl#1 PWS#1
DMSO DMSO MG132 Baf
MAP2
Ctrl#2 PWS#2
DMSO DMSO MG132 Baf
MAP2
Ctrl#3 PWS#3
DMSO DMSO MG132 Baf
MAP2
Ctrl#2 PWS#2
DMSO DMSO MG132 Baf
TUBB3
Ctrl#1 PWS#1
DMSO DMSO MG132 Baf
TUBB3
Ctrl#3 PWS#3
DMSO DMSO MG132 Baf
TUBB3
Ctrl#1 PWS#1
DMSO DMSO MG132 Baf
GAPDH
Ctrl#2 PWS#2
DMSO DMSO MG132 Baf
GAPDH
Ctrl#3 PWS#3
DMSO DMSO MG132 Baf
GAPDH

Supplemental Figure 1

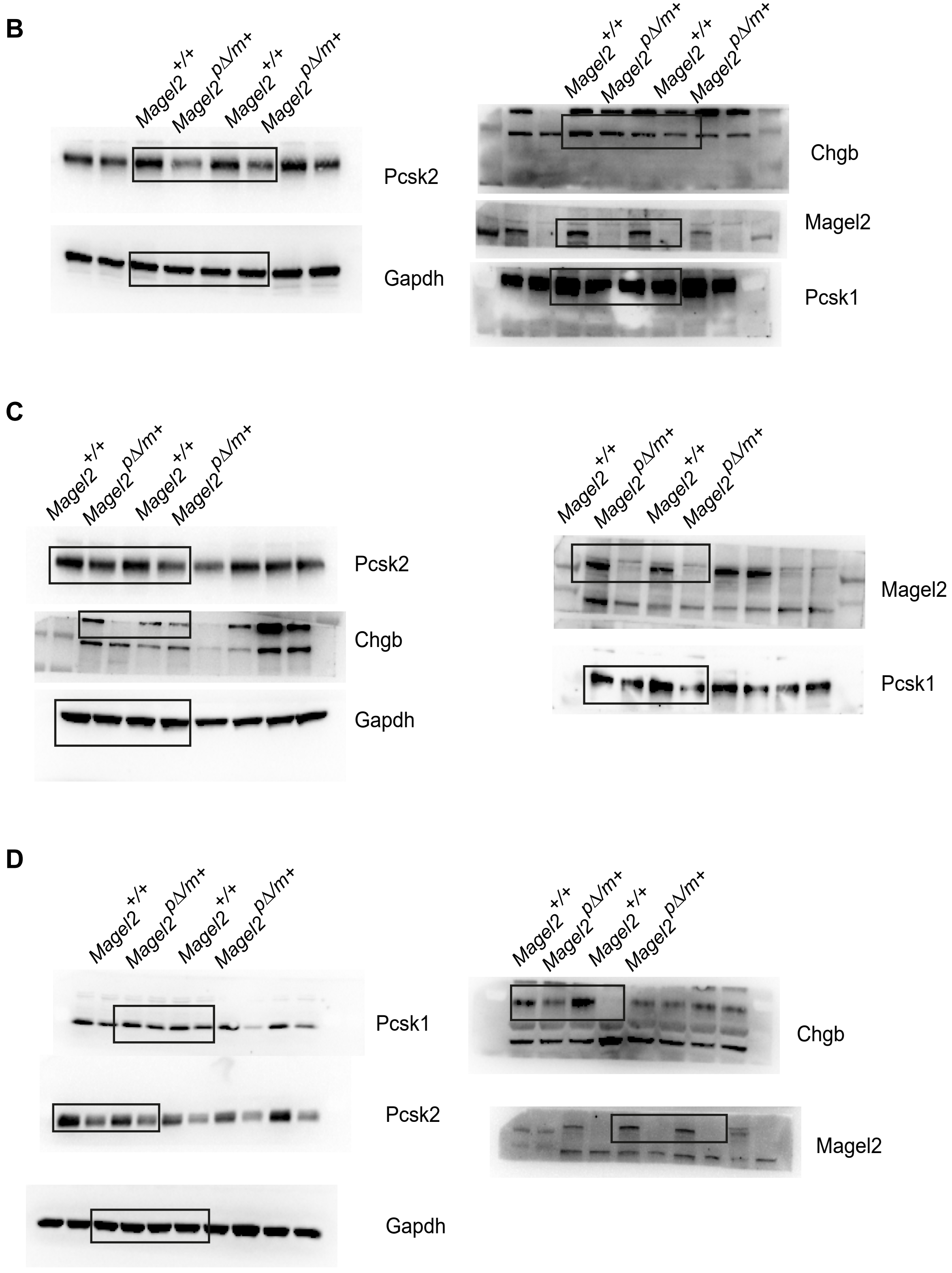

Supplemental Figure 4

B

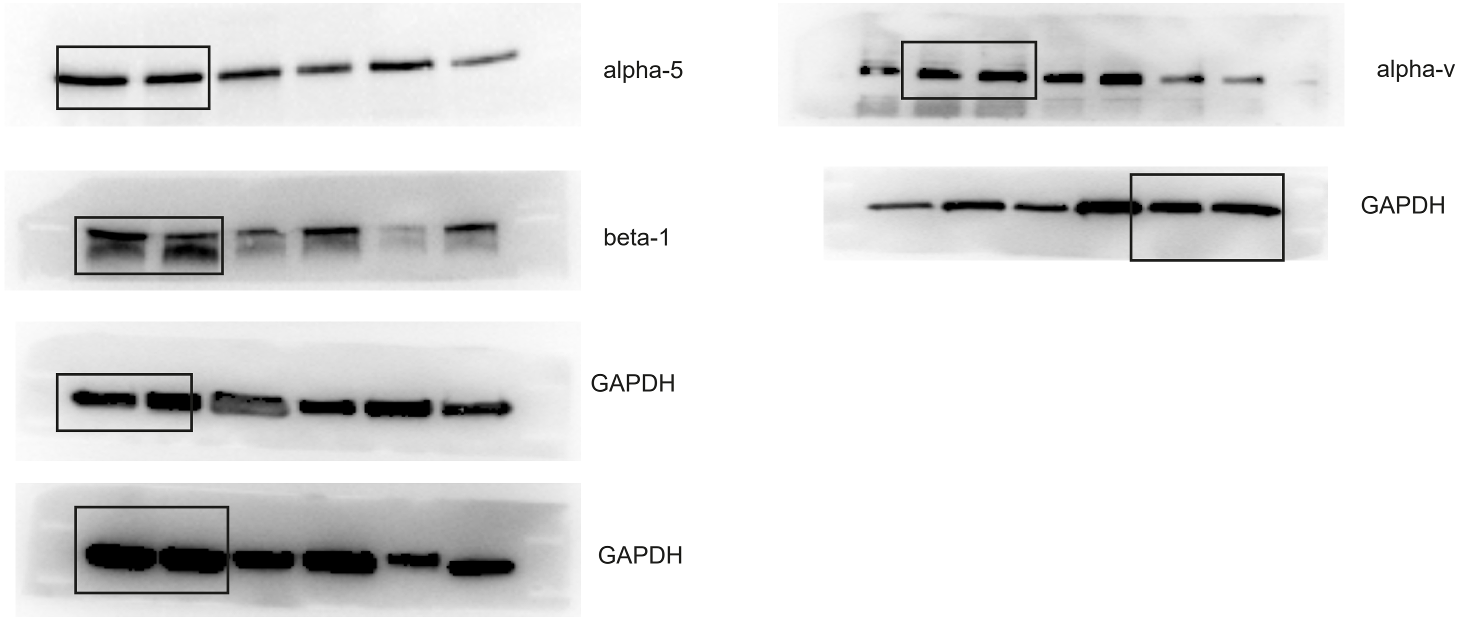

Supplemental Figure 6

A

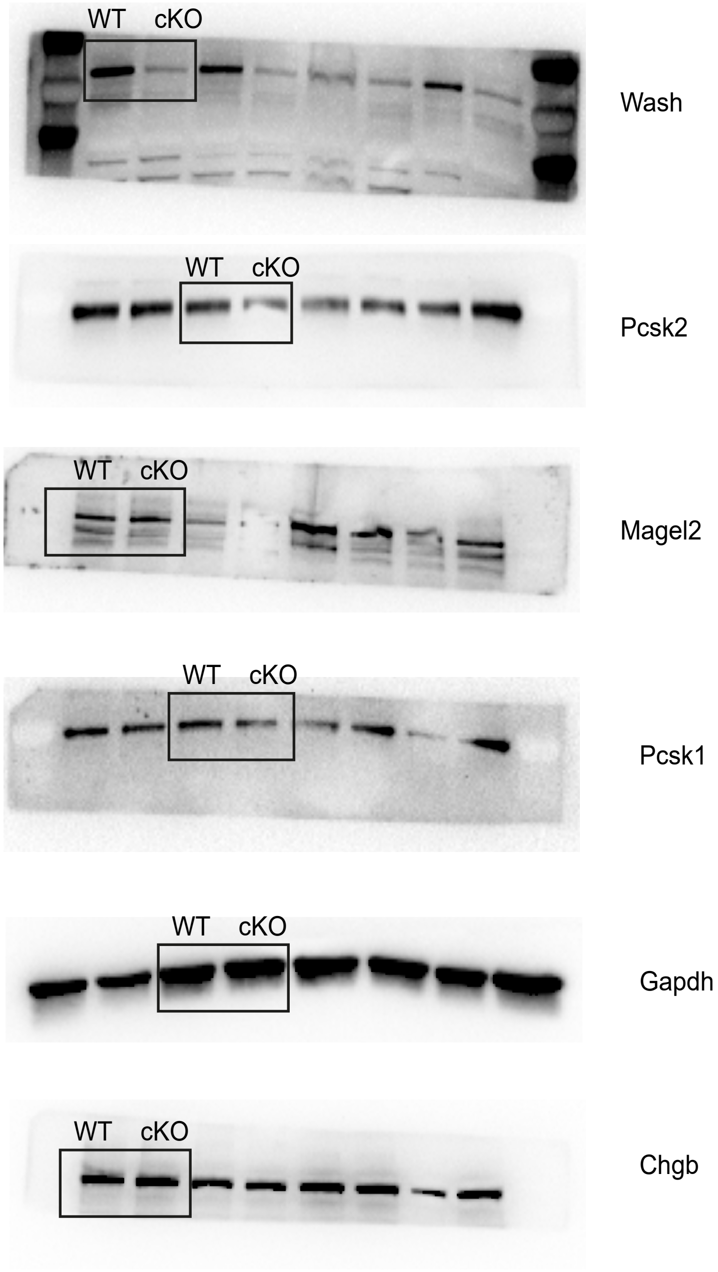

Supplement: Supplemental data [file jciinsight-5-138576-s020.pdf]
